# Ultrafast photophysics of an orange–red thermally activated delayed fluorescence emitter: the role of external structural restraint†

**DOI:** 10.1039/d4sc00460d

**Published:** 2024-04-03

**Authors:** Yixuan Gao, Yaxin Wang, Zilong Guo, Yan Wan, Zheng Xue, Yandong Han, Wensheng Yang, Xiaonan Ma

**Affiliations:** a Institute of Molecular Plus, Tianjin University Tianjin 300072 P. R. China xiaonanma@tju.edu.cn zilong.guo@tju.edu.cn; b Engineering Research Center for Nanomaterials, Henan University Kaifeng 475004 P. R. China; c College of Chemistry, Beijing Normal University Beijing 100875 P. R. China

## Abstract

The application of thermally activated delay fluorescence (TADF) emitters in the orange–red regime usually suffers from the fast non-radiative decay of emissive singlet states (*k*^S^_NR_), leading to low emitting efficiency in corresponding organic light-emitting diode (OLED) devices. Although *k*^S^_NR_ has been quantitatively described by energy gap law, how ultrafast molecular motions are associated with the *k*^S^_NR_ of TADF emitters remains largely unknown, which limits the development of new strategies for improving the emitting efficiency of corresponding OLED devices. In this work, we employed two commercial TADF emitters (TDBA-Ac and PzTDBA) as a model system and attempted to clarify the relationship between ultrafast excited-state structural relaxation (ES-SR) and *k*^S^_NR_. Spectroscopic and theoretical investigations indicated that S_1_/S_0_ ES-SR is directly associated with promoting vibrational modes, which are considerably involved in electronic–vibrational coupling through the Huang–Rhys factor, while *k*^S^_NR_ is largely affected by the reorganization energy of the promoting modes. By restraining S_1_/S_0_ ES-SR in doping films, the *k*^S^_NR_ of TADF emitters can be greatly reduced, resulting in high emitting efficiency. Therefore, by establishing the connection among S_1_/S_0_ ES-SR, promoting modes and *k*^S^_NR_ of TADF emitters, our work clarified the key role of external structural restraint for achieving high emitting efficiency in TADF-based OLED devices.

## Abstract 2

Non-radiative decay of orange–red TADF emitters are strongly associated with excited-state structural relaxation through promoting vibrational modes, for which external structural restraint is critical for achieving high OLED emitting efficiency.

## Graphical abstract

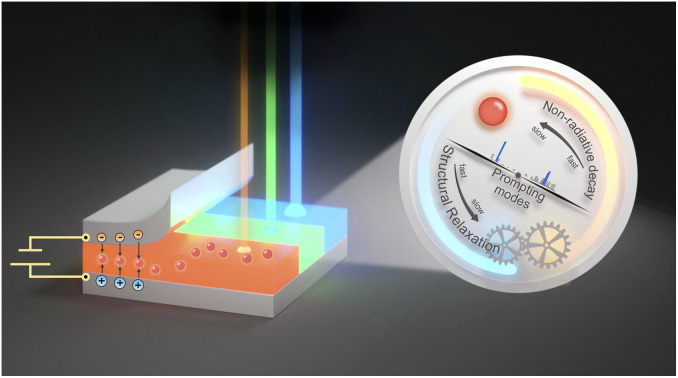

## Introduction

Organic light-emitting diode (OLED) technology is becoming increasingly attractive due to its great potential for ultra-high definition (UHD) displays.^1–5^ As a key factor in the performance of OLED devices, external quantum efficiency (*η*_EQE_) is described as the ratio between the number of emitted photons and number of injected carriers. In most cases, the *η*_EQE_ of OLED devices can be expressed as the product of several contributing terms:^6,7^

1 *η*_EQE_ = *γ* × *η*_EUE_ × *Φ*_F_ × *η*_out_

where the carrier balance factor (*γ*) and output coupling factor (*η*_out_) are associated with the device design and fabrication, respectively, while exciton utilization efficiency (*η*_EUE_) and fluorescent quantum yield (*Φ*_F_) are regarded as intrinsic properties of the emitters and are associated with corresponding excited-state processes. Spin statistics show that the recombination of injected electrons and holes leads to 25% singlet and 75% triplet states in OLED emitting layers, respectively,^8,9^ while *η*_EUE_ gives the fraction of excited states that can decay to the ground state radiatively. Since T_1_ → S_0_ decay is spin-forbidden, *η*_EUE_ is largely determined by the quantum yield (*Φ*_RISC_) of the reverse intersystem crossing (RISC, T_1_ → S_0_, with rate constant of *k*_RISC_), which competes with other T_1_ relaxation routes (radiative *k*^T^_R_ and non-radiative *k*^T^_NR_, Fig. S1†):^10,11^2
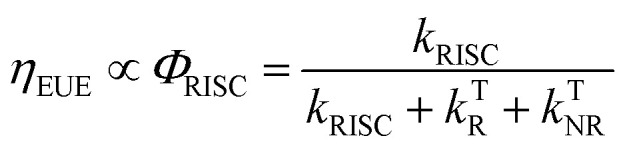


Enormous efforts have been made towards designing thermally activated delayed fluorescence (TADF) emitters with minimized singlet–triplet energy gap (Δ*E*_ST_) and enhanced spin–orbit coupling (SOC) to enable thermal conversion of T_1_ → S_1_, which can potentially push *η*_EUE_ to ∼100%.^12–15^ Since the *k*_RISC_ of TADF emitters is usually on a time scale of 10^3^–10^5^ s^−1^, *η*_EUE_ is generally associated with slow dynamics of TADF emitters, which has been intensively investigated.^14,16–18^

In addition to the efficient utilization of current-generated triplet states, the *Φ*_F_ of TADF emitters greatly affects the *η*_EQE_ of the corresponding OLED devices, which describes the ratio of singlet (S_1_) states that can be radiatively relaxed to the S_0_ state, *i.e.*, competing plausible relaxation channels for the S_1_ state (Fig. S1†):^19–21^3
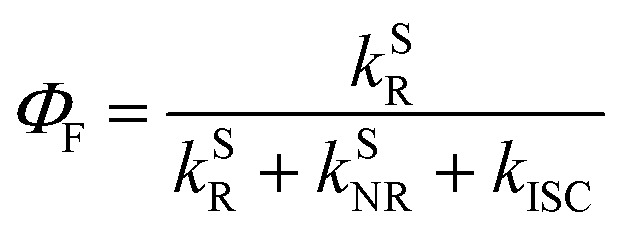
In addition, *Φ*_F_ includes a contribution from the S_1_ state directly generated by injected carriers (*Φ*_PF_, prompt fluorescence) and converted from the T_1_ state (*Φ*_DF_, delayed fluorescence):

4 *Φ*_F_ = *Φ*_PF_ + *Φ*_DF_

For organic TADF emitters, *k*_ISC_ is usually slow (10^7^–10^8^ s^−1^),^16,22^ for which the presence of rapid non-radiative decay is a key factor that can significantly reduce the *Φ*_F_ of TADF emitters. In a weak coupling regime, the rate constant of the S_1_ state non-radiative decay (*k*^S^_NR_) with energy gap (Δ*E*_S_1_–S_0__) and electronic coupling (*C*) can be described as^23–25^5
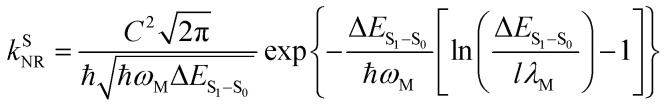
where *ω*_M_ and *λ*_M_ represent the frequency and reorganization energy contribution of the promoting vibrational mode, while *l* is the number of involved vibrational modes. By assuming *l* = 1, eqn (5) can be simplified to the famous energy gap law with the molecule-specific parameter *γ* as^21,25–27^6
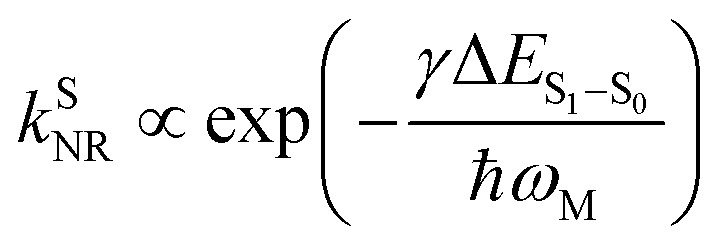
which indicates that *k*^S^_NR_ increases exponentially with the reduction of the S_1_ → S_0_ energy gap (Δ*E*_S_1_–S_0__), leading to challenges in the design of long-wavelength TADF emitters with high *Φ*_F_.^28–31^ As a result, reported TADF emitters in the blue to green regime largely exhibit considerably high *Φ*_F_,^32–34^ while the *η*_EQE_ of the corresponding OLED devices are primarily affected by RISC. In contrast, the small Δ*E*_S_1_–S_0__ of orange–red TADF emitters leads to fast *k*^S^_NR_ competing with the radiative decay of the S_1_ state, resulting in a low *Φ*_F_ and subsequently an unsatisfactory *η*_EQE_ for OLED devices, although up to 100% *η*_EUE_ can be expected *via* efficient RISC.^19,28,35–37^

**Scheme 1:**
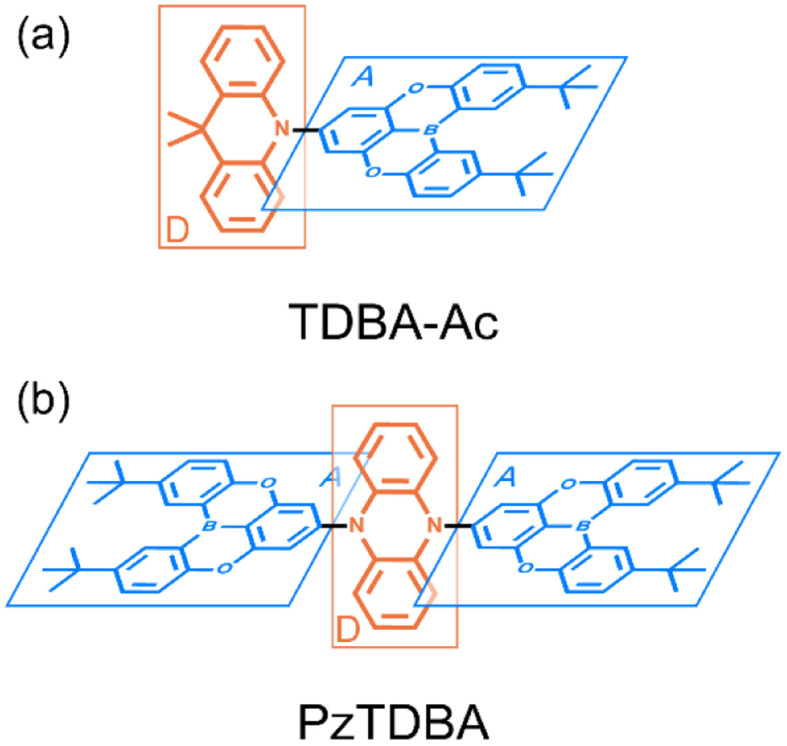
Illustrated chemical structure of investigated TADF emitters: (a) TDBA-Ac, D–A type; (b) PzTDBA, A–D–A type. The charge donors and acceptors are displayed in blue and red, respectively.

Recently, enormous efforts have been made towards developing long-wavelength TADF-based OLED devices with a high *η*_EQE_,^28,35^ in which the low *Φ*_F_ of TADF emitters is usually regarded as the main obstacle.^28,38–40^ Therefore, one can expect opportunities to further improve the *η*_EQE_ of TADF-based long-wavelength OLED devices by somehow effectively slowing down the *k*^S^_NR_ rate.^41–44^ Recently, Kwon and co-workers reported an acceptor–donor–acceptor (ADA) type TADF emitter (PzTDBA, Scheme 1) and realized >30% of the *η*_EQE_ of orange–red OLED devices.^45^ Although the excited-state mechanism that is responsible for the high *η*_EQE_ is still ambiguous, the reported ∼100% *Φ*_F_ of PzTDBA in doping films indicates that non-radiative decay of the S_1_ state is nearly terminated, which seems to violate the well-known energy gap law (eqn (6)). Therefore, understanding the underlying ultrafast photophysics that is associated with the slow *k*^S^_NR_ of PzTDBA might provide inspiration for designing high-performance TADF emitters in the long-wavelength regime.

In this work, the ultrafast photophysics of an orange–red TADF emitter (PzTDBA, ADA-type) and its DA-type analogue (TDBA-Ac, deep-blue) was investigated using femtosecond transient absorption (fs-TA), time-resolved fluorescence (tr-FL) and theoretical vibrational analysis.^46^ Compared with the one-step D–A twisting of the TDBA-Ac emitter, PzTDBA exhibits two-step S_0_/S_1_ excited-state structural relaxation (ES-SR), *i.e.* fast D–A twisting and slow planarization of the Pz group. The promoting vibrational modes associated with the S_0_/S_1_ ES-SR motions of the TDBA-Ac and PzTDBA emitters were theoretically identified, which dominate the electronic-vibrational coupling (EVC) of the S_1_ state. Meanwhile, promoting modes contribute to fast *k*^S^_NR_ through the corresponding reorganization energy contribution (*λ*_M_). In doping films, the S_0_/S_1_ ES-SR motions of PzTDBA are suppressed by external structural restraint, which greatly slows down *k*^S^_NR_ and leads to ∼100% *Φ*_F_. Our work established the connection among the S_0_/S_1_ ES-SR, promoting modes and S_1_ state non-radiative decay and indicated the key role of medium rigidity in improving the emitting efficiency of TADF emitters, which should provide inspiration for the future development of TADF emitters.

## Results and discussion

### Low-lying excited states and steady state spectra

**Fig. 1:**
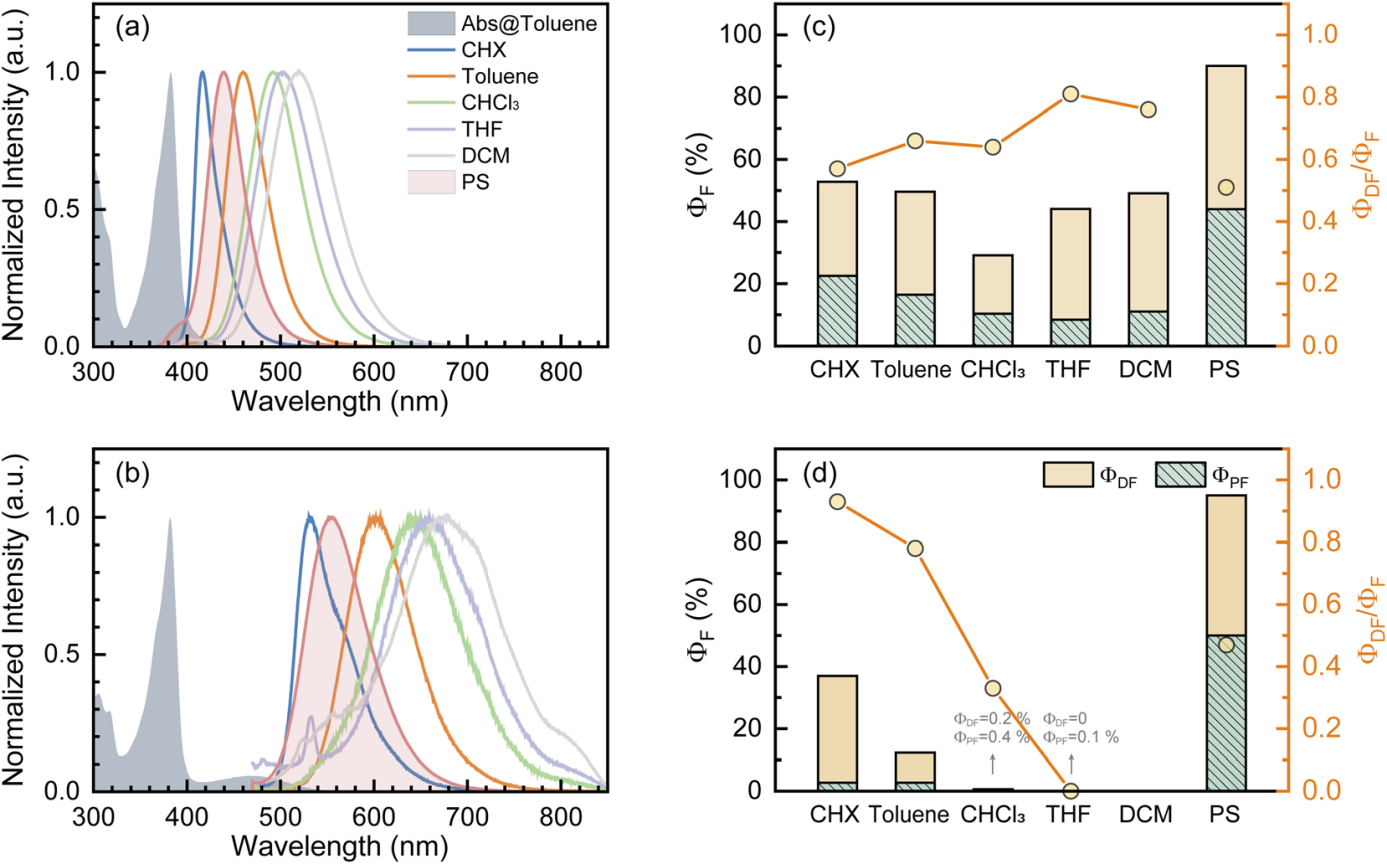
The steady UV/vis absorption and fluorescence spectra of TDBA-Ac (a) and PzTDBA (b) in various solutions and PS doping films. Measured total fluorescence quantum yield (*Φ*_F_), corresponding prompt (*Φ*_PF_) and delayed (*Φ*_DF_) contribution, as well as the related contribution of the delayed component (*Φ*_DF_/*Φ*_F_) of TDBA-Ac (c) and PzTDBA (d).

Fundamental photophysics of TDBA-Ac and PzTDBA emitters were first investigated in solvents with different polarities (Table S1†). In OLED devices, TADF emitters are usually doped in a wide-bandgap organic semiconductor as the host. To avoid multi-photon excitation of the host in fs-TA experiments, we prepared doping films (2 wt%) of TADF emitters by employing polystyrene (PS) as the host, and the polarity of the PS medium (*ε* = 2.6–2.7, Δ*f* = 0.03) was determined to be comparable with toluene (Fig. S2†).^47,48^ The UV/Vis absorption and fluorescence spectra of the TDBA-Ac and PzTDBA emitters in various solutions and films (2 wt%) can be seen in Fig. 1 and S3.† The TD-DFT (M06-2X/6-311G**, PCM = toluene) calculated vertical low-lying singlet and triplet states (S_1_–S_3_, T_1_–T_3_) are listed in Table S2† with the visualized distribution of frontier orbitals (HOMO−2 to LUMO+2) shown in Table S3.† The lowest-lying S_1_ states of TDBA-Ac and PzTDBA are dominated by the H → L transition, corresponding to the charge transfer state (noted as ^1^CT_DA_) with low oscillator strength, which can be seen as weak absorption of TDBA-Ac (400–450 nm) and PzTDBA (420–540 nm). Upon UV excitation, solvatochromism was observed for TDBA-Ac and PzTDBA, indicating that the fluorescence emission originated from the lowest-lying ^1^CT_DA_ state.^49,50^ Intriguingly, ∼1000 cm^−1^ (TDBA-Ac) and ∼1400 cm^−1^ (PzTDBA) blue-shifted emissions were observed in PS doping films compared to in toluene (comparable polarity), indicating the presence of multifaceted interactions, which are discussed below.

The T_1_ state of TDBA-Ac was identified as a local excited state on the acceptor (noted as ^3^LE_A_) while the T_1_ excitation of PzTDBA was localized on the donor (^3^LE_D_), which allows direct T_1_ → S_1_ RISC (^3^LE_D/A_ → ^1^CT_DA_).^51,52^ Meanwhile, the T_2_ states of TDBA-Ac and PzTDBA were recognized as charge transfer states (^3^CT_DA_). With identical orbital wavefunction to the ^1^CT_DA_ state, the corresponding T_2_ → S_1_ RISC (^3^CT_DA_ → ^1^CT_DA_) is forbidden.^51,52^ By further confirming the CT/LE nature of low-lying singlet/triplet states through natural transition orbital (NTO) analysis (Fig. S4 and S5†) and hole–electron analysis (Table S4†),^53,54^ the T_1_ → S_1_ (^3^LE_D/A_ → ^1^CT_DA_) transition was assigned as the accessible RISC channel that is responsible for the delayed fluorescence of TDBA-Ac and PzTDBA.

The thermally accessible Δ*E*_ST_ is critical for RISC, while estimating Δ*E*_ST_ through the vertical excitation energy of S_1_ and T_1_ states was reported to be unreliable.^55^ Thus, we further optimized the S_1_ and T_1_ geometry of the TDBA-Ac and PzTDBA emitters to estimate adiabatic 
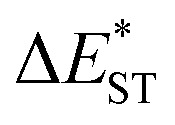
. As listed in Table 1, TDBA-Ac and PzTDBA exhibit 
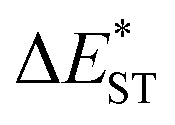
 of ∼0.3 eV and ∼0.26 eV in low-polarity media (CHX and TOL, Δ*f* < 0.02), respectively, facilitating RISC for harvesting T_1_ states with moderate 〈S_1_|*Ĥ*_SO_|T_1_〉. However, increasing to medium polarity (in DCM, Δ*f* = 0.22) leads to enlarged 
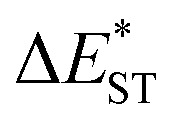
 (>0.33 eV) for TDBA-Ac and PzTDBA with nearly unchanged 〈T_1_|*Ĥ*_SO_|S_1_〉, which might explain the reduced RISC rate (*k*_RISC_). Note that the vertical (Δ*E*_ST_) and adiabatic 
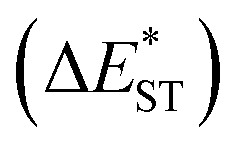
 singlet-triplet energy gap does not have a certain magnitude relationship as it depends on multiple factors, such as the different ES-SR in the S_1_/T_1_ states and steepness of the corresponding potential energy surface (PES), which are simplified as schematic diagrams (Fig. S6†). The total *Φ*_F_ and corresponding prompt/delayed (*Φ*_PF_/*Φ*_DF_) contribution of TDBA-Ac and PzTDBA were further measured in different solutions and PS doping films. As illustrated in Fig. 1c, TDBA-Ac exhibits nearly solvent-independent *Φ*_F_ (30–50%). With increasing solvent polarity, the solvation of the ^1^CT_DA_ (S_1_) state and unchanged energy level of the ^3^LE_A_ (T_1_) state resulted in reduced Δ*E*_ST_,^50,56^ which is consistent with the observed increasing of the *Φ*_DF_ contribution (*Φ*_DF_/*Φ*_F_, Fig. 1c). Upon optical excitation, S_1_ → T_1_ → S_1_ is the only feasible channel for delayed fluorescence,^17,38,57^ and increased *Φ*_DF_/*Φ*_F_ might correspond to higher *Φ*_ISC_ and *Φ*_RISC_. In contrast, the *Φ*_F_ of PzTDBA rapidly decreased from 37% in non-polar CHX to undetectably low in polar mediums, which implies different relaxation of the photo-generated S_1_ state. Intriguingly, TDBA-Ac and PzTDBA exhibit nearly 100% *Φ*_F_ and an evenly divided contribution of *Φ*_PF_ and *Φ*_DF_ (*Φ*_DF_/*Φ*_F_ ≈ 50%) in PS doping films, indicating that the S_1_ state decay is dominantly radiative, while all ISC-generated T_1_ states can be harvested through subsequent RISC. Considering the importance of *Φ*_F_, resolving the photophysics behind the fully radiative relaxation of orange–red PzTDBA in doping film would be of high interest.

**Table 1:** TDDFT calculated vertical (*E*) and adiabatic (E*) excitation energy of the lowest-lying (S_1_, T_1_) states and corresponding energy gaps (Δ*E*_ST_, 
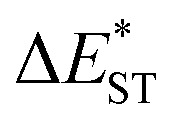
) of TDBA-Ac and PzTDBA in different mediums using PCM as the model; SOC matrix elements for ISC (〈S_1_|*Ĥ*_SO_|T_1_〉) and RISC (〈T_1_|*Ĥ*_SO_|S_1_〉) were calculated by using the linear response approach

	TDBA-Ac			PzTDBA		
	CHX	TOL	DCM	CHX	TOL	DCM
*E*(S_1_)/eV	3.617	3.626	3.667	3.130	3.142	3.207
*E*(T_1_)/eV	3.250	3.251	3.252	3.015	3.014	3.010
Δ*E*_ST_/eV	0.367	0.375	0.415	0.115	0.128	0.197
E*(S_1_)/eV	3.474	3.482	3.509	2.792	2.804	2.861
E*(T_1_)/eV	3.173	3.173	3.174	2.539	2.538	2.531
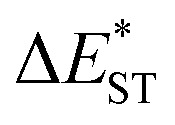 /eV	0.301	0.309	0.335	0.253	0.266	0.330
〈S_1_|*Ĥ*_SO_|T_1_〉/cm^−1^	0.338	0.339	0.294	0.016	0.018	0.058
〈T_1_|*Ĥ*_SO_|S_1_〉/cm^−1^	0.341	0.341	0.341	0.053	0.052	0.058

### tr-FL and excited-state relaxation

The time-resolved fluorescence (tr-FL) measurements of TDBA-Ac and PzTDBA were further performed by time-correlated single-photon counting (TCSPC) upon excitation at *λ*_ex_ = 400 nm.

The resulting traces were fitted as the sum of prompt (*τ*_PF_) and delayed (*τ*_DF_) components (Fig. S7, S8† and Table 2). Combining with measured *Φ*_PF_ and *Φ*_DF_, the rate constants for ISC (S_1_ → T_1_, *k*_ISC_) and RISC (T_1_ → S_1_, *k*_RISC_) as well as corresponding *Φ*_ISC_ and *Φ*_RISC_ were estimated (Table 2). With calculated SOC matrix elements (〈S_1_|*Ĥ*_SO_|T_1_〉 and 〈T_1_|*Ĥ*_SO_|S_1_〉), we attempted to reproduce the experimentally extracted values of *k*_ISC_ and *k*_RISC_ using the thermal vibration correlation function (TVCF)^16,54,58^ and semi-classical Marcus^59,60^ approaches. However, as shown in Table S5,† TVCF and Marcus approaches failed to describe *k*_ISC_ and *k*_RISC_, which might be attributed to the ignorance of non-Condon effects, such as Herzberg–Teller coupling and spin–vibronic coupling.^61–63^ Meanwhile, we noticed that calculation errors for the *k*_ISC_ of PzTDBA are much more significant than for TDBA-Ac, which might imply higher structural flexibility of PzTDBA and will be discussed in detail below. By further estimating the experimental values of *k*^S^_R_, *k*^S^_NR_ and *k*^T^_NR_ (Table 2), the quantitative contribution of the plausible relaxation channels was calculated for the S_1_ and T_1_ states.

**Table 2:** Measured photophysical data for the TDBA-Ac and PzTDBA emitters in N_2_-saturated solutions and PS doping films

	TDBA-Ac				PzTDBA			
	aCHX	aTOL	aDCM	bPS	aCHX	aTOL	aDCM	bPS
*τ*_PF_/ns	6.72	21.35	36.15	14.12	8.70	23.95	13.62	38.25
*τ*_DF_/μs	0.15	0.14	0.48	1.21	0.10	0.29	0.23	1.10
*Φ*_F_	0.53	0.49	0.47	0.90	0.37	0.13	<0.01	0.95
*Φ*_PF_	0.23	0.16	0.11	0.44	0.03	0.03	<0.01	0.50
*Φ*_DF_	0.30	0.33	0.36	0.46	0.34	0.10	0	0.45
f *Φ*_ISC_	0.57	0.67	0.78	0.51	0.93	0.78	0	0.47
g *Φ*_RISC_	0.39	0.39	0.43	0.82	0.35	0.10	0	0.90
c *k*_PF_/10^7^ s^−1^	14.88	4.69	2.77	7.14	11.49	4.17	7.35	2.63
d *k* ^S^_R_/10^6^ s^−1^	33.48	7.70	3.05	31.43	3.10	1.13	—	13.16
e *k* ^S^_NR_/10^6^ s^−1^	30.29	7.82	3.16	3.49	5.28	8.02	73.46	0.69
d *k*_ISC_/10^7^ s^−1^	8.50	3.14	2.15	3.65	10.66	3.25	—	1.25
c *k*_DF_/10^6^ s^−1^	3.57	3.54	1.01	0.74	3.63	0.43	—	0.86
d *k*_RISC_/10^5^ s^−1^	18.75	17.57	4.98	6.69	13.42	0.52	—	8.20
d *k* ^T^_NR_/10^5^ s^−1^	31.50	32.55	9.60	4.49	35.91	4.24	—	4.53

a Concentration of 10^−5^ M.

b Doping concentration of 2 wt%.

c Calculated by *k*_PF_ = 1/*τ*_PF_ and *k*_DF_ = 1/*τ*_DF_.

d Rate constants *k*^S^_R_, *k*_ISC_, *k*_RISC_ and *k*^T^_NR_ were calculated using the method described by Adachi *et al*.^10,19^

e Calculated using *k*^S^_NR_ = *k*_PF_ − *k*^S^_R_ − *k*_ISC_.

f Calculated using *Φ*_ISC_ = *k*_ISC_/(*k*_ISC_ + *k*^S^_R_ + *k*^S^_NR_).

g Calculated using *Φ*_RISC_ = *Φ*_DF_/(1 − *Φ*_PF_).

**Fig. 2:**
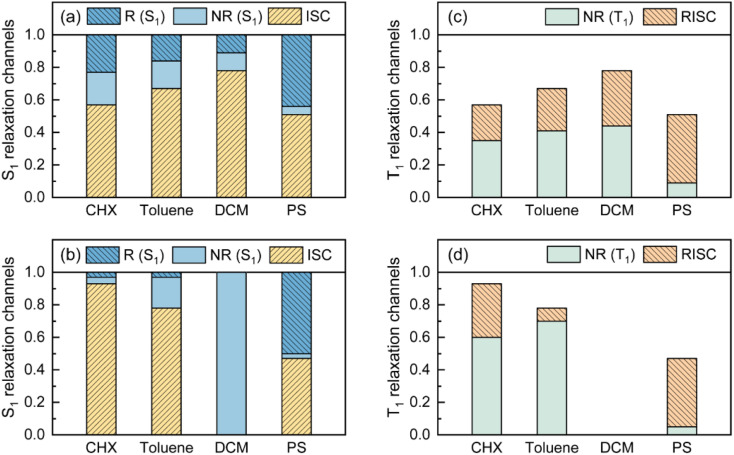
Visualized contribution of ISC (S_1_ → T_1_), radiative (R, S_1_ → S_0_) and non-radiative (NR, S_1_ → S_0_) decay of S_1_ state for TDBA-Ac (a) and PzTDBA (b) in various solutions and PS doping films. Contribution of RISC (T_1_ → S_1_) and non-radiative (NR, T_1_ → S_0_) decay of T_1_ state for TDBA-Ac (c) and PzTDBA (d) in various solutions and PS doping films, radiative decay of T_1_ state was ignored due to low contribution.

As visualized in Fig. 2, the S_1_ relaxation of TDBA-Ac is dominated by the slightly increased ISC with increased solvent polarity, while the ISC-generated T_1_ states can be converted to S_1_ with nearly identical *Φ*_RISC_, which leads to the nearly unchanged *Φ*_F_ of TDBA-Ac in CHX (0.53), TOL (0.49) and DCM (0.47). In contrast, although ISC still dominates the S_1_ decay of PzTDBA in CHX and TOL, the generated T_1_ states largely decay non-radiatively to S_0_ rather than thermally converting to S_1_*via* RISC, for which a low *Φ*_F_ was observed in CHX (0.37) and TOL (0.13). In high-polarity DCM, the S_1_ decay of PzTDBA is predominately occupied by the non-radiative path to S_0_, leading to an undetectable *Φ*_F_ (<0.01), which might be attributed to the exponentially increased *k*^S^_NR_ described by band-gap law with the reducing of the S_1_ → S_0_ energy gap (Δ*E*_S_1_–S_0__).

Intriguingly, TDBA-Ac and PzTDBA exhibit nearly identical pattern of S_1_ and T_1_ decay in PS doping films, which is largely differed with observation in solutions. The S_1_ decay of TDBA-Ac and PzTDBA in PS doping films are dominated by equally divided radiative decay (S_1_ → S_0_) and ISC (S_1_ → T_1_), while ISC-generated T_1_ states can be further converted to S_1_ through efficient RISC (*Φ*_RISC_ > 0.9). Due to negligible role of non-radiative path in both S_1_ (<5%) and T_1_ (<10%) state decay, TDBA-Ac and PzTDBA exhibit unexpectedly high *Φ*_F_ in PS doping films, which agrees with the reported high *η*_EQE_ of corresponding OLED devices.^45,46^ However, such high *Φ*_F_ (0.95) of PzTDBA observed in PS doping film seems to be inconsistent with the energy gap law, implying unique photophysics by which non-radiative decay of S_1_ state can be greatly suppressed.

### fs-TA and structural relaxation

The fs-TA signal upon UV excitation (*λ*_ex_ = 320 nm) of TDBA-Ac and PzTDBA in CHX solution and PS doping film were recorded by broadband probe (*λ*_probe_ = 350–750 nm) with delay times up to 7.0 ns, and the temporal resolution of fs-TA was measured to be ∼90 fs. As shown in Fig. S9 and S10,† the measured fs-TA signals of TDBA-Ac and PzTDBA are contributed by negative ground-state bleaching (GSB) in the *λ*_probe_ = 350–400 nm regime, and the positive excited-state absorption (ESA) band extended in the longer wavelength region up to *λ*_probe_ = 750 nm.

Upon UV excitation, high-lying S_*n*_ states can be populated, followed by rapid internal conversion (IC, S_*n*_ → S_1_) to the long-lived S_1_ state, which explains the dramatically reshaped ESA in the 1 ps delay time.^64,65^ For the subsequent delay times of 1 ps to 1 ns, fs-TA evolution visualizes wavepacket motion that highly depends on the topology of S_1_ PES.^41,66–68^ For instance, the role of the excited-state structural relaxation (ES-SR) in the S_1_ state decay of DA- and DAD/ADA-type TADF emitters has been intensively reported.^69–73^ Suffering from the poor structural sensitivity of UV/Vis fs-TA, S_1_/S_0_ ES-SR usually leads to minimized alternation of the ESA shape, which is consistent with the observed fs-TA of TDBA-Ac and PzTDBA for 1 ps to 1 ns delay times.^41,74^

**Fig. 3:**
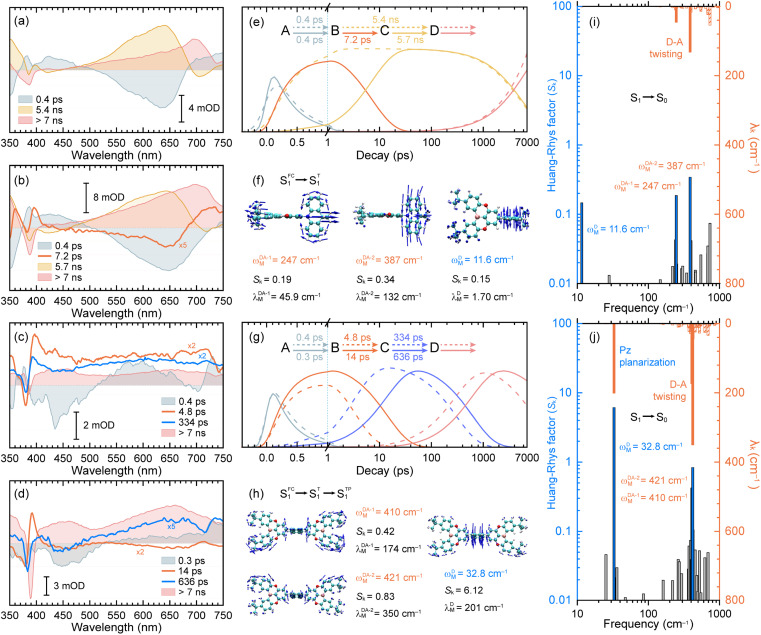
The decay-associated spectra (DAS) extracted from the fs-TA data of TDBA-Ac in CHX solution (a) and PS doping film (b) as well as PzTDBA in CHX solution (c) and PS doping film (d). Fitted concentration evolution of TDBA-Ac (e) and PzTDBA (g) in CHX solution (dashed lines) and PS doping film (solid lines). Promoting vibrational modes of TDBA-Ac (f) and PzTDBA (h) with large contribution to the HR factors. Calculated HR factors and reorganization energy contribution of each vibrational mode of TDBA-Ac (i) and PzTDBA (j) for the S_1_ → S_0_ transition in CHX solution. The dominant modes displayed in (f) and (h) are highlighted correspondingly.

For characterizing S_1_/S_0_ ES-SR, the geometries of TDBA-Ac and PzTDBA in the S_0_, S_1_ and T_1_ states were optimized using DFT and TD-DFT approaches. The resulting structures are illustrated in Table S6,† while total reorganization energies (*λ*_S_0_→S_1__ and *λ*_S_1_→S_0__) were calculated for evaluating the ES-SR of the TDBA-Ac and PzTDBA emitters together with the root of the mean squared displacement (RMSD_S_1_/S_0__) between the S_0_ and S_1_ states (Fig. S11†). As listed in Table 3, the calculated values of *λ*_S_0_→S_1__, *λ*_S_1_→S_0__ and RMSD_S_1_/S_0__ indicate a more pronounced ES-ER of PzTDBA in the S_1_ state than TDBA-Ac, which obviously cannot be attributed to structural extension (DA to ADA structure) as the total reorganization energy and RMSD are not additive. To evaluate the contribution of each of the molecular fragments of the TDBA-Ac and PzTDBA emitters to the ES-SR, several critical structural parameters were defined, as illustrated in Fig. S12† and measured (Table 3) for the S_0_ and S_1_ states. Upon vertical excitation, TDBA-Ac and PzTDBA initially remain in S_0_ geometry in the Franck–Condon (FC) region, following by ES-SR until reaching the global minimum of S_1_ PES, *i.e.* optimized S_1_ geometry. For TDBA-Ac, S_1_/S_0_ ES-SR featured a slight increase in the D–A twisting angle *β* from 89.82° (S^FC^_1_) to 92.00° (S^T^_1_), while the dihedral angles of the Ac (*α*) and TDBA (*γ*) framework bending remained nearly unchanged in the S_1_ state decay. In contrast, ADA-type PzTDBA exhibits higher flexibility than that of TDBA-Ac. In addition to the fast motion of the D–A twisting angle (*β*) from 79.72°/79.06° (S^FC^_1_) to 89.02°/91.43° (S^T^_1_), framework planarization of the center donor (Pz) was observed as the dihedral angle (*α*) increased from 164.57° (S^T^_1_) to 180.00° (S^TP^_1_). The simultaneous S_0_–S_1_ state changing of *α* and *β* angles may correspond to a two-step S_1_/S_0_ ES-SR (S^FC^_1_ → S^T^_1_ → S^TP^_1_), which has been widely reported,^75,76^*i.e.* fast D–A twisting (S^FC^_1_ → S^T^_1_) followed by slow framework planarization (S^T^_1_ → S^TP^_1_). Target analysis was further performed to acquire quantitative information on S_1_/S_0_ ES-SR that may play a key role in the S_1_ state relaxation. By including three or four sequential decay processes, measured fs-TA data can be well-reproduced by the displayed decay-associated spectra (DAS) of each decay components and concentration evolution of each transient species (Fig. 3), while species-associated spectra (SAS) can be seen in Fig. S13 and S14.†

**Table 3:** DFT and TD-DFT (M06-2X, 6-311G**, PCM = toluene) calculated optimal geometric parameters of TDBA-Ac and PzTDBA in the S_0_, S_1_ and T_1_ states, as well as the calculated total reorganization energy and RMSD associated with the S_1_ state ES-SR^a^

	TDBA-Ac			PzTDBA		
	*α (°)	**β (°)	***γ (°)	*α (°)	**β_1_/*β*_2_ (°)	***γ_1_/*γ*_2_ (°)
S_0_ geometry	176.50	89.82	11.36	164.57	79.72/79.06	11.56/10.99
S_1_ geometry	175.45	92.00	12.12	180.00	89.02/91.43	11.65/11.37
T_1_ geometry	176.57	90.82	4.77	180.00	88.60/91.33	11.40/11.40
*λ*_S_0_→S_1__ (cm^−1^)	1161			2790		
*λ*_S_1_→S_0__ (cm^−1^)	1273			2552		
RMSD_S_1_/S_0__ (Å)	0.053			0.241		

a *Bending dihedral angle of donor (Ac/Pz); **donor–acceptor twisting angle; ***bending dihedral angle of acceptor (TDBA).

The initial decay (A → B) with 300–400 fs time constant was accompanied by considerable ESA reshaping, corresponding to rapid IC (S_*n*_ → S_1_). The fs-TA was subsequently dominated by S_1_/S_0_ ES-SR until the wavepacket reached the global minimum of S_1_ PES. For PzTDBA, highlighted two-step S_1_/S_0_ ES-SR (B → C → D, *i.e.* S^FC^_1_ → S^T^_1_ → S^TP^_1_) predicted by calculated S_0_ and S_1_ geometries was observed, featuring a slight ESA reshaping. The observed fast (4.8 ps in CHX, S^FC^_1_ → S^T^_1_) and slow (334 ps in CHX, S^T^_1_ → S^TP^_1_) steps correspond to D–A twisting (*β*_1_/*β*_2_ angles) and Pz planarization (*α* angle), respectively.

For TDBA-Ac, one-step S_1_/S_0_ ES-SR was observed (7.2 ps, S^FC^_1_ → S^T^_1_) in the PS doping film, corresponding to the D–A twisting (β angle), which is comparable with the fast S_1_/S_0_ ES-SR step of PzTDBA. However, S_1_/S_0_ ES-SR of TDBA-Ac was unobservable in CHX, which might be attributed to a less pronounced S_0_ → S_1_ structural change than for PzTDBA, *i.e.* nearly unchanged *β* angle in the S_0_ (89.82°) and S_1_ (92.00°) states.

Intriguingly, the extracted time constants of D–A twisting (S^FC^_1_ → S^T^_1_, 14.0 ps) and Pz planarization (S^T^_1_ → S^TP^_1_, 636 ps) of PzTDBA in the PS doping film are 2–3 times slower than corresponding S_1_/S_0_ ES-SR steps in CHX (Fig. 3g and S15†), implying a higher potential barrier for S_1_ PES, which is consistent with our fs-TA observations for multiple-resonance emitters.^77^ Moreover, slow S_1_ isomerization of azo-benzene embedded in polymer films was reported previously, attributing to external structural restraint from polymer micro-networks.^78^

As discussed above, the fluorescence emission of PzTDBA was strongly quenched (*Φ*_F_ = 0.37 in CHX) in solutions due to the presence of fast non-radiative decay, while a high *Φ*_F_ (>0.95) in the PS doping film may be feasible if non-radiative S_1_ → S_0_ decay can be greatly suppressed. Our fs-TA data revealed higher S_1_/S_0_ ES-SR barriers of PzTDBA in PS doping films than in CHX, which might imply the presence of an underlying association between S_1_/S_0_ ES-SR and the non-radiative decay of the S_1_ state, *i.e.* restrained S_1_/S_0_ ES-SR leads to suppressed non-radiative decay. Conversely, *k*^S^_NR_ was described to be highly correlated with promoting vibrational modes by the energy gap law (eqn (5)).^21,23^ Thus, we attempted to unveil the underlying relationship among the S_1_/S_0_ ES-SR, promoting modes and non-radiative decay of the S_1_ state.

### Vibrational analysis and non-radiative decay

The energy gap law (eqn (5)) clearly describes how promoting vibrational modes affect *k*^S^_NR_ through their vibrational frequency (*ω*_M_) and reorganization energy (*λ*_M_), while promoting modes are vibrational modes that are considerably involved in the EVC of the S_1_ state through a pronounced Huang-Rhys (HR) factor (*S*_k_).^24,79,80^ We further calculated the *S*_k_ and *λ*_k_ contribution of each of the vibrational modes involved in the S_1_ → S_0_ transition (Fig. 3i and j), in which the promoting modes associated with the *k*^S^_NR_ of TDBA-Ac and PzTDBA are illustrated in Fig. 3f and h, respectively.

As shown in Fig. 3j and h, three promoting modes were identified for the S_1_ → S_0_ transition of PzTDBA due to considerable *S*_k_. The modes at 410 cm^−1^ (*ω*^DA-1^_M_) and 421 cm^−1^ (*ω*^DA-2^_M_) correspond to symmetric and asymmetric D–A twisting and exhibit considerable reorganization energy contributions (*λ*^DA-1^_M_ = 174 cm^−1^ and *λ*^DA-2^_M_ = 350 cm^−1^), which are associated with the fast S_1_/S_0_ ES-SR step observed on fs-TA of PzTDBA, *i.e.* S^FC^_1_ → S^T^_1_ with the D–A twisting motion. For TDBA-Ac (Fig. 3i and f), the corresponding S_1_/S_0_ ES-SR of D–A twisting is associated with promoting modes at *ω*^DA-1^_M_ = 247 cm^−1^ and *ω*^DA-2^_M_ = 387 cm^−1^ with substantially lower *λ*^DA-1^_M_ (45.9 cm^−1^) and *λ*^DA-2^_M_ (132 cm^−1^), which is consistent with its rigid structure predicted theoretically, *i.e.* less pronounced S_1_/S_0_ ES-SR than PzTDBA.

Meanwhile, S_1_ → S_0_ decay of PzTDBA features a promoting mode at *ω*^D^_M_ = 32.8 cm^−1^ with a surprisingly high HR factor (*S*_k_ = 6.12) and considerable reorganization energy (*λ*^D^_M_ = 201.1 cm^−1^), corresponding to the bending motion of the Pz framework, which is clearly associated with the observed slow S_1_/S_0_ ES-SR step of PzTDBA, *i.e.* S^T^_1_ → S^TP^_1_. Intriguingly, this particular mode was also observed for TDBA-Ac at *ω*^D^_M_ = 11.6 cm^−1^ with a much lower HR factor (*S*_k_ = 0.15) and a two orders of magnitude lower reorganization energy contribution (*λ*^D^_M_ = 1.70 cm^−1^) than that of PzTDBA, indicating that it was excluded from the S_1_ → S_0_ decay of TDBA-Ac. As a result, the second S_1_/S_0_ ES-SR step (S^T^_1_ → S^TP^_1_) was absent from the fs-TA signal of TDBA-Ac.

Furthermore, we investigated the influence of medium polarity on the promoting modes of the TDBA-Ac and PzTDBA emitters. As shown in Fig. S16,† for PzTDBA in DCM solution, an extra promoting mode at 15.4 cm^−1^ was observed with considerable *S*_k_ but ignorable *λ*_k_, which might be less associated with the non-radiative decay of S_1_ state PzTDBA. In contrast, a promoting mode at 12.4 cm^−1^ was observed for TDBA-Ac with considerable *S*_k_ (8.71) and *λ*_k_ (108.23 cm^−1^) in DCM solution, which is very different from the case of TDBA-Ac in low-polarity solvents, indicating that the S_1_/S_0_ ES-SR of TDBA-Ac (DA-type) is more evidentially coupled with charge transfer than PzTDBA (ADA-type).

**Fig. 4:**
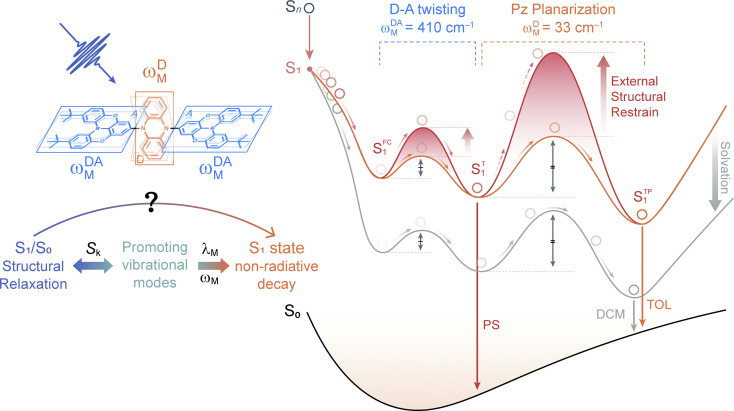
Simplified energetic diagram illustrating two-step ES-SR (S^FC^_1_ → S^T^_1_ and S^T^_1_ → S^TP^_1_) of PzTDBA in low-polarity solution (orange line, TOL), high-polarity solution (cyan line, DCM) and PS doping film (red line). The double arrows represent the potential barriers of D–A twisting (S^FC^_1_ → S^T^_1_) and Pz planarization (S^T^_1_ → S^TP^_1_).

As discussed above, the one- (TDBA-Ac, S^FC^_1_ → S^T^_1_) and two-step (PzTDBA, S^T^_1_ → S^TP^_1_) S_1_/S_0_ ES-SR are directly associated with promoting vibrational modes that are considerably involved in the EVC of the S_1_ state through their HR factor. Meanwhile, promoting modes contribute to the *k*^S^_NR_ of the S_1_ state through corresponding *λ*_M_, which implies that S_1_/S_0_ ES-SR can significantly affect *k*^S^_NR_ (and subsequently *Φ*_F_) through the promoting vibrational modes (Fig. 4). For instance, the one-step S_1_/S_0_ ES-SR of TDBA-Ac is associated with vibrational modes with low *S*_k_ and *λ*_M_, while the promoting modes associated with the two-step S_1_/S_0_ ES-SR of PzTDBA have much higher *S*_k_ and *λ*_M_. As a result, the non-radiative channel plays a minor role in the S_1_ decay of structurally rigid TDBA-Ac, while the emission of structurally flexible PzTDBA is severely hampered by the fast non-radiative decay of the S_1_ state. Furthermore, the two-step S_1_/S_0_ ES-SR motions of PzTDBA are greatly suppressed in the PS doping films due to the external structural restraint, for which the S_1_ non-radiative decay associated with the promoting modes might be correspondingly weakened, leading to the greatly improved *Φ*_F_ of PzTDBA in the doping films. In this sense, the external structural restraint for S_1_/S_0_ ES-SR motions might be critical for achieving a high *η*_EQE_ for TADF-based OLED devices.

To directly verify the influence of the external structural restraint on the S_1_ non-radiative decay of PzTDBA, we measured temperature-dependent emission spectra of PzTDBA in polyethylene oxide (PEO) doping films (Fig. S18†). With a glass transition temperature (*T*_g_) of 220 K, PEO provides a “softer” microenvironment than PS (*T*_g_ = 373 K) and can be further “softened” by increasing the temperature.^81,82^ As a TADF emitter, PzTDBA was expected to be more fluorescent at higher temperatures due to faster RISC. However, we observed clear fluorescence quenching with increasing temperature in the 297–347 K range, indicating that non-radiative decay was enhanced in the medium with reduced external structural restraint, which is consistent with our analysis described above.

Note that we cannot perform vibrational analysis for TADF emitters with the presence of the external structural restraint, but we speculate that the *λ*_M_ of the promoting modes of PzTDBA might be greatly reduced due to the external structural restraint, in agreement with slowed S_1_/S_0_ ES-SR motions in the doping films, especially the Pz bending mode (*ω*^D^_M_) associated with the slow S_1_/S_0_ ES-SR step (S^T^_1_ → S^TP^_1_) might be terminated. As a result, S^T^_1_ instead of S^TP^_1_ might dominate the emission of PzTDBA in the PS doping films (see Fig. 4), which explains the observed blue-shifted emission in the PS doping film compared with the case in toluene solution, which has comparable polarity to the PS medium. Meanwhile, the emission of PzTDBA in solutions mainly originates from S^TP^_1_ due to the accessible barrier of S^T^_1_ → S^TP^_1_, which thus suffers from the plague of non-radiative decay (Fig. 4).

Last but not least, in addition to the S_1_ state, we noticed that TDBA-Ac and PzTDBA exhibit a suppressed non-radiative decay channel of the T_1_ state in the PS doping films compared to in solutions (Fig. 2c and d), which inspired us to consider the role of T_1_/S_0_ ES-SR in the non-radiative decay of the T_1_ state. As shown in Fig. S17,†TDBA-Ac exhibits several vibrational modes with considerable *S*_k_ in the region <100 cm^−1^, but the low *λ*_M_ implies that ES-SR may not be strongly associated with non-radiative T_1_ → S_0_ decay. In contrast, the promoting modes of PzTDBA at 32.9 cm^−1^ and 421/410 cm^−1^ contribute considerable *λ*_M_, while pronounced T_1_/S_0_ ES-SR was indicated (Table 3), including Pz bending and D–A twisting. Therefore, it might be plausible that the non-radiative T_1_ → S_0_ decay is similarly associated with two T_1_/S_0_ ES-SR motions, because the non-radiative T_1_ → S_0_ decay of PzTDBA was strongly suppressed in the PS doping film due to the external structural restraint. However, unlike S_1_ relaxation, without direct spectroscopic evidence of T_1_/S_0_ ES-SR in different media, verifying this hypothesis requires further investigation.

## Conclusions

To summarize, we performed a comprehensive investigation of the photophysics of two TADF emitters, TDBA-Ac (DA-type, blue light) and PzTDBA (ADA-type, orange–red light), using fs-TA, tr-FL and theoretical approaches. Compared with the one-step S_1_/S_0_ ES-SR (S^FC^_1_ → S^T^_1_) of TDBA-Ac, the S_1_ state decay of PzTDBA is dominated by two steps, S_1_/S_0_ ES-SR (S^FC^_1_ → S^T^_1_ → S^TP^_1_), while greatly slowed S_1_/S_0_ ES-SR motions of PzTDBA were observed in the PS doping films due to the external structural restraints. Vibrational analysis indicated that the S_1_/S_0_ ES-SR motions are directly associated with the promoting modes that are considerably involved in the EVC of the S_1_ state through their own *S*_k_, while the promoting modes contribute to fast *k*^S^_NR_*via λ*_M_. In doping films, the external structural restraint leads to suppressed S_1_/S_0_ ES-SR and reorganization energy contribution of the promoting modes, resulting in slowed *k*^S^_NR_ and a highly fluorescent S_1_ state that is favorable for OLED application. With TDBA-Ac and PzTDBA as model systems, we established the connection among the S_0_/S_1_ ES-SR, promoting modes and *k*^S^_NR_ of TADF emitters, which indicated the key role of the external structural restraint in obtaining high *Φ*_F_ TADF emitters. Our work also provides a new direction for OLED design, and it might be necessary to take the rigidity of host materials used for the emitting layer into consideration to avoid emission quenching through fast non-radiative decay associated with the S_1_/S_0_ ES-SR of TADF emitters, especially for TADF emitters with a relatively small band gap, which might be a useful reference for future OLED application.

## Data availability

All experimental and calculational data are available from the corresponding author upon reasonable request.

## Author contributions

Yixuan Gao: conceptualization, methodology, investigation, data curation, formal analysis, visualization, and writing – original draft; Yaxin Wang: methodology, data curation, formal analysis, and validation; Zilong Guo: investigation, visualization, project administration, and supervision; Yan Wan: methodology and resources; Zheng Xue and Yandong Han: resources and project administration; Wensheng Yang: supervision, resources, and funding acquisition; Xiaonan Ma: conceptualization, formal analysis, funding acquisition, supervision, and writing – review and editing.

## Conflicts of interest

The authors declare no competing financial interest.

## Supplementary Material

SC-015-D4SC00460D-s001
