# Supplementary material for: Ultrafast photophysics of an orange–red thermally activated delayed fluorescence emitter: the role of external structural restraint
Source: Chem Sci. 2024 Apr 3;15(17):6410–20. doi: 10.1039/d4sc00460d (PMC11062098; doi:10.1039/d4sc00460d)
Supplement: SC-015-D4SC00460D-s001 [file SC-015-D4SC00460D-s001.pdf]

Electronic Supplementary Material (ESI) for Chemical Science.
This journal is © The Royal Society of Chemistry 2024

Supporting Information

Ultrafast Photophysics of Orange–Red Thermally Activated Delayed Fluorescence Emitter: Role of External Structural Restrain

Yixuan Gao,[†] *Yaxin Wang*,[†] *Zilong Guo*,[*,†] *Yan Wan*,[⊥] *Zheng Xue*,[‡] *Yandong Han*,[‡] *Wensheng Yang*[‡,†] and *Xiaonan Ma*[*,†]

[†] Institute of Molecular Plus, Tianjin University, Tianjin 300072, P. R. China;

[‡] Engineering Research Center for Nanomaterials, Henan University, Kaifeng 475004, P. R. China;

[⊥] College of Chemistry, Beijing Normal University, Beijing 100875, P. R. China.

*xiaonanma@tju.edu.cn; zilong.guo@tju.edu.cn

Contents

Section	Content	Page
S1	Experimental method	3
S2	Photophysics of TADF emitters	9
	Fig. S1	9
S3	Information on mediums	10
	Table S1	10
	Fig. S2	10
S4	Steady spectra	11
	Fig. S3	11
S5	Low-lying excited-states	12
	Table S2	12
	Table S3	13
	Fig. S4	14
	Fig. S5	15
	Table S4	16
S6	Energy gap between S_1 and T_1	17
	Fig.S6	17
S7	Time-resolved Fluorescence	18
	Fig. S7	18
	Fig. S8	19
S8	Calculating k_{ISC} and k_{RISC}.	20
	Table.S5	20
S9	fs-TA measurements	21
	Fig. S9	21
	Fig. S10	22
	Fig. S11	23
	Fig. S12	24
	Table S6	24
	Fig. S13	25
	Fig. S14	26
	Fig. S15	27
S10	Vibrational analysis	28
	Fig. S16	28
	Fig. S17	29
S11	Temperature-dependent fluorescence spectra	30
	Fig. S18	30

Section S1. Experimental method

Chemical and materials.

The TDBA-Ac and PzTDBA samples were purchased from Luminescence Technology Corp. and used without further purification. All involved organic solvents are HPLC grade and used as received. For fabricating PS doping films of TDBA-Ac and PzTDBA, PS solutions were firstly prepared by dissolving 1 g PS in 11.5 mL toluene. The resulted PS solution (2 mL) was then mixed with 3 mg TDBA-Ac or PzTDBA powder, corresponding to 2wt% doping concentration. The two-step dissolving was performed with stirring (400 rpm) at room temperature. The PS solution of TDBA-Ac or PzTDBA were further spin-coated (1000 rpm, 1 min) on 1 mm thick quartz substrates with 1000 rpm/s acceleration, resulting in doping films with absorbance of 0.2–0.3 at excitation wavelength of TDBA-Ac and PzTDBA.

Spectroscopic experiments.

Steady and tr-FL. The steady-state UV/Vis absorption and photoluminescence spectra were recorded on U-3900 (Hitachi, Japan) spectrophotometer and F-4700 (Hitachi, Japan) fluorescence spectrometer, respectively. The fluorescence quantum yield (Φ_F) of solution was determined by using Coumarin 153 (Φ_F = 0.38, in EtOH) as a reference.[1] For oxygen-free measurements, solutions were bubbled with nitrogen flow for 15 min before measurements. The Φ_F of PS doping films were measurements by an integrating sphere with CW excitation at 365 nm. The fluorescence time traces of solution and doping films were recorded with a time correlated single-photon counting (TCSPC) spectrometer (PTI

Quanta Master 800, HORIBA) equipped with a 330 nm nano-LED excitation source, which leads to an instrument response function (IRF) value of 0.6 ns.

fs-TA. The fs-TA measurements were performed by using home-built ultrafast pump-probe spectrometer upon 320 nm optical excitation. Briefly, a commercial 1 kHz Ti:sapphire laser system delivered ~40 fs pulses centered at 800 nm. The ~60 fs excitation pulses (λ_{pump} = 320 nm) were derived from the second-harmonic output of a collinear optical parametric amplifier. Approximately 0.1–0.3 μJ pulse energy was measured at the sample position. The broadband UV/Vis probe pulses were generated by focusing a small portion of the Ti:sapphire laser fundamental into a linearly moving CaF_2 window, resulting in a white-light spectrum between 350 nm and 750 nm. Pump and probe beams were spatially overlapped in a quartz cuvette with 500 μm sample thickness or in PS films directly, optical density of sample was controlled to 0.1–0.3 at 320 nm. The polarization of pump and probe beams were set to magic angle of 54.7°. After passing the sample, the probe pulses were dispersed in a grating spectrometer and detected by a linear Si detector array. The measured TA data were evaluated via target analysis with software package Glotaran[2] based on the R-package TIMP.[3]

Theoretical calculation.

Electronic structure and wavefunction analysis. All electronic structure calculation of TDBA-Ac and PzTDBA were performed using Gaussian 09[4] and 16[5] software packages. The geometric structure of investigated emitters was optimized on both ground (S_0), singlet (S_1) and triplet (T_1) excited states at M06-2X/6-311G** level while no imaginary frequency was found by frequency analysis. The vertical (ΔE_{ST}) and adiabatic (ΔE_{ST}*)

energy gaps between singlet (S_1) and triplet (T_1) excited states were estimated by single point and geometric optimization calculation, respectively. The frontier orbital distribution of investigated emitters were rendered by using the VMD 1.9.3 program.[6] The natural transition orbitals (NTOs) and hole-electron analysis of corresponding excited states were performed by using Multiwfn program,[7] which leads to parameters for quantifying charge transfer of corresponding excited states. The S_r index (hole–electron overlap) was calculated as follow:

$$S_{\mathrm{r}}\left(r\right)=\sqrt{\rho^{hole}(r)\rho^{ele}(r)} \tag{1}$$

where ρ^{hole} (r) and ρ^{ele} (r) denote spatial distribution of hole and electron wavefunction, respectively.

Vibrational analysis. The Huang-Rhys (HR) factor (S_k) and reorganization energy contribution (λ_k) of each vibrational modes were calculated by using MOMAP software on basis of frequency analysis of corresponding states. We further estimated reorganization energy contribution (λ_k) of each vibrational modes between S_1 and S_0 states with the harmonic oscillator approximation:

$$\lambda_k=\sum_k\frac{1}{2}\omega_k^2\Delta Q_k^2 \tag{2}$$

in which λ_k can be calculated by the corresponding frequency (ω_k) and vibrational displacement (ΔQ_k), while ΔQ_k can be estimated as a linear combination of internal coordinates, *i.e.*

$$\Delta Q_k = \sum_j \zeta_{kj} \Delta D_j \tag{3}$$

where ΔD_j represents displacement along the internal coordinate j of the equilibrium position in the $S_1 \rightarrow S_0$ transition.

The HR factor (S_k) of each vibrational mode k can be calculated with its frequency (ω_k) and displacement (ΔQ_k).

$$S_k = \frac{1}{2\hbar} \omega_k \Delta Q_k^2 \tag{4}$$

Spin-orbital coupling. The SOC matrix elements between singlet (S_1) and triplet (T_1) excited states were calculated by using the linear-response LR methods implemented in PySOC program,[8] which has been included in MOMAP software.[9] The SOC Hamiltonian can be approximately calculated as

$$\hat{H}_{\mathrm{SO}} \approx \sum_i^{N_e} \zeta(r_i) \hat{\mu}_{\mathrm{L}} \hat{\mu}_{\mathrm{S}} \tag{5}$$

where μ_L and μ_S represent magnetic moment operators resulting from orbital and spin angular momentum with SOC constant $\zeta(r_i)$.

Structural relaxation. To further quantify S_1/S_0 structural relaxation, the root of the mean of squared displacement (RMSD) between S_0 (x_i, y_i, z_i) and S_1 (x_i', y_i', z_i') state was calculated in various solution.

$$\mathrm{RMSD} = \sqrt{\frac{1}{N} \sum_{\mathrm{i}}^{N} \left[(x_{\mathrm{i}} - x_{\mathrm{i}}')^2 + (y_{\mathrm{i}} - y_{\mathrm{i}}')^2 + (z_{\mathrm{i}} - z_{\mathrm{i}}')^2 \right]} \tag{6}$$

by summing over all atoms i = 1, ..., N.

ISC and RISC rate. The ISC and RISC rate of investigated TADF emitters were calculated by using the thermal vibration correlation function (TVCF) method implemented in MOMAP software, in which geometric displaced, distorted and Duschinsky rotation effects between corresponding electronic states were taken into consideration. Specifically, non-radiative ISC ($S_1 \rightarrow T_1$) and RISC ($T_1 \rightarrow S_1$) rate can be calculated with SOC term and corresponding TVCF $\rho_{ISC}(t, T)$ with form of $\mathrm{Tr}[e^{-i\tau_\mathrm{f}\hat{H}_\mathrm{f}}e^{-i\tau_\mathrm{i}\hat{H}_\mathrm{i}}]$ by

$$k_{\mathrm{ISC}} = \frac{1}{\hbar^2}\left\langle \Psi_\mathrm{f} \middle| \hat{H}_{\mathrm{SO}} \middle| \Psi_\mathrm{i} \right\rangle \int_{-\infty}^{\infty} \mathrm{d}t \left[\mathrm{e}^{\mathrm{i}\omega_{\mathrm{if}}t} Z_\mathrm{i}^{-1} \rho_{\mathrm{ISC}}(t, T) \right] \quad (7)$$

Moreover, ISC/RISC kinetics can be also considered in the framework of Marcus theory,[10] which could be expressed as:

$$k_{\mathrm{ISC}} = \frac{2\pi}{\hbar} \left| H_{\mathrm{SO}}^{\mathrm{ISC}} \right|^2 (4\pi\lambda k_\mathrm{B}T)^{-\frac{1}{2}} \exp\left(\frac{-E_A^{\mathrm{ISC}}}{k_\mathrm{B}T}\right) \quad (8)$$

$$k_{\mathrm{RISC}} = \frac{2\pi}{\hbar} \left| H_{\mathrm{SO}}^{\mathrm{RISC}} \right|^2 (4\pi\lambda k_\mathrm{B}T)^{-\frac{1}{2}} \exp\left(\frac{-E_A^{\mathrm{RISC}}}{k_\mathrm{B}T}\right) \quad (9)$$

where ħ is the reduced Planck constant, k_B is the Boltzmann constant, T is the temperature, λ is the reorganization energy, and E_A is the activation energy to reach the crossing seam. In the case of simple parabolic PESs with equal force constants, which is a crucial assumption of Marcus theory, E_A can be analytically expressed as:

$$E_A^{\mathrm{ISC}} = \frac{(\Delta E_{\mathrm{ST}} + \lambda)^2}{4\lambda} \quad (10)$$

For RISC transition, the activation energy can be approximately described as $E_A^{RISC} = E_A^{ISC}+\Delta E_{ST}$.

Reference

1 G. Jones, W. R. Jackson, C. Y. Choi and W. R. Bergmark, *J. Phys. Chem.*, 1985, **89**, 294–300.
2 I. H. M. Van Stokkum, D. S. Larsen and R. Van Grondelle, *BBA Bioenerg.,* 2004, **1657**, 82–104.
3 K. M. Mullen and I. H. M. V. Stokkum, *J. Stat. Soft.*, 2007, **18**, 3.
4 Frisch, M. J.; Trucks, G. W.; Schlegel, H. B.; Scuseria, G. E.; Robb, M. A.; Cheeseman, J. R.; and Scalmani, G.; Barone, V.; Petersson, G. A.; Nakatsuji, H.; et al., Gaussian 09, Revision D.01 (version Gaussian 09, Revision D.01) Gaussian, Inc., Wallingford CT 2013.
5 Frisch, M. J.; Trucks, G. W.; Schlegel, H. B.; Scuseria, G. E.; Robb, M. A.; Cheeseman, J. R.; and Scalmani, G.; Barone, V.; Petersson, G. A.; Nakatsuji, H.; et al., Gaussian 16, Revision A.03 (version Gaussian 16, Revision A.03) Gaussian, Inc., Wallingford CT 2016.
6 W. Humphrey, A. Dalke and K. Schulten, *Journal of Molecular Graphics*, 1996, **14**, 33–38.
7 T. Lu and F. Chen, *J. Comput. Chem.*, 2012, **33**, 580–592.
8 X. Gao, S. Bai, D. Fazzi, T. Niehaus, M. Barbatti and W. Thiel, *J. Chem. Theory Comput.*, 2017, **13**, 515–524.
9 Y. Niu, W. Li, Q. Peng, H. Geng, Y. Yi, L. Wang, G. Nan, D. Wang and Z. Shuai, *Molecular Physics*, 2018, **116**, 1078–1090.
10 N. Aizawa, Y. Harabuchi, S. Maeda and Y.-J. Pu, *Nat. Commun.*, 2020, **11**, 3909.

Section S2. Photophysics of TADF emitters.

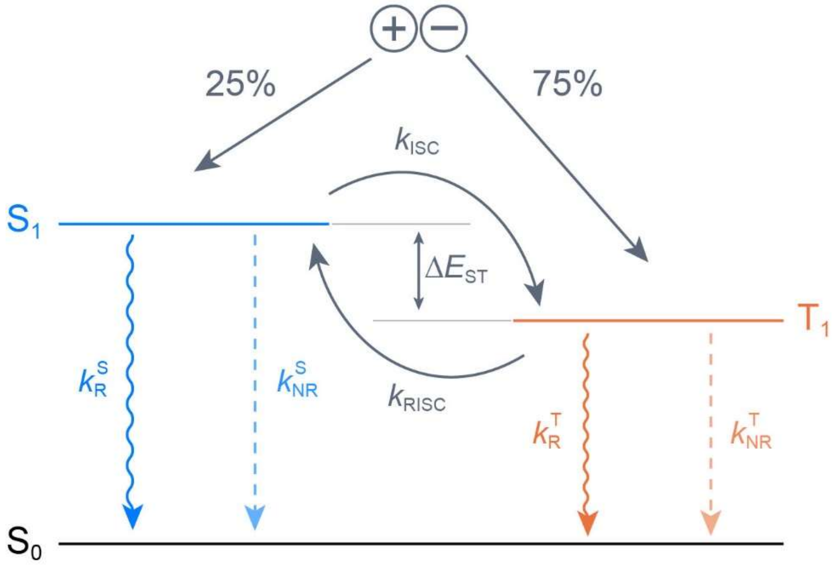

Fig. S1 The singlet and triplet excitons are formed after electron–hole recombination in a singlet: triplet ratio of 1:3. k_{ISC}, ISC rate; k_{RISC}, RISC rate; $k_R{}^S$, radiative of singlet excitons; $k_R{}^T$, radiative of triplet excitons. $k_{NR}{}^S$, non-radiative of singlet excitons; $k_{NR}{}^T$, non-radiative of triplet excitons.

Section S3. Information on mediums.

Table S1 Physical properties of involved solvents.

Solvent		Chemical formula	ε	n	Δ*f*
cyclohexane	CHX	C_6H_{12}	2.023	1.424	0
toluene	TOL	C_7H_8	2.379	1.494	0.014
chloroform	/	$CHCl_3$	4.810	1.443	0.149
tetrahydrofuran	THF	C_4H_8O	7.580	1.405	0.210
dichloromethane	DCM	CH_2Cl_2	8.930	1.421	0.218

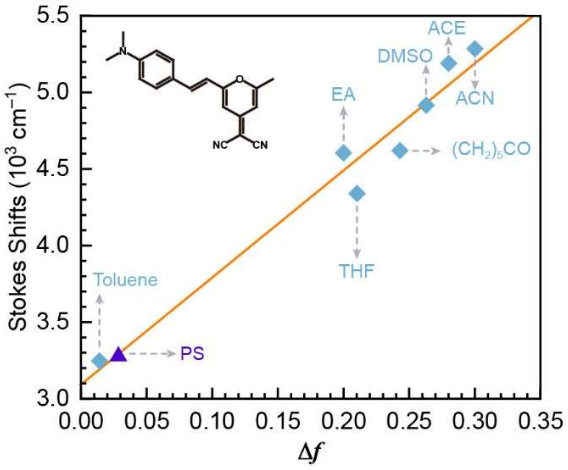

Fig. S2 The Lippert-Mataga plotting of laser dye 4-(Dicyanomethylene)-2-methyl-6-(4-dimethylaminostyryl)-4H-pyran (DCM) in varies of solvents (blue square), the polarity of PS (purple triangle) is observed by the fitted relationship.

Section S4. Steady spectra.

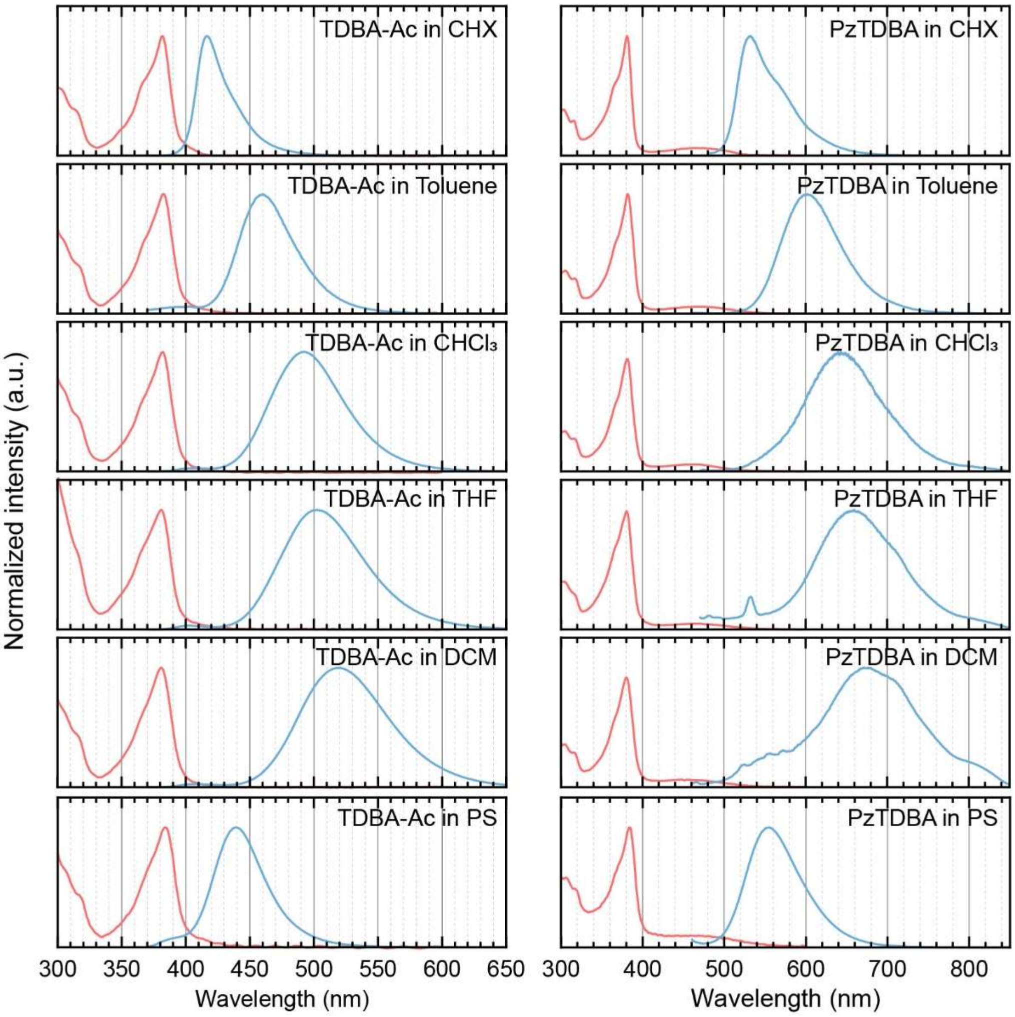

Fig. S3 The UV/Vis absorption (red line) and photoluminescence spectra (blue line) of TDBA-Ac and PzTDBA in varies of solution and PS film.

Section S5. Low-lying excited-states.

Table S2 The TD-DFT (M06-2X, 6-311g**, PCM = toluene) calculated vertical excited-states (S_1 ~ S_3, T_1 ~ T_3) of TDBA-Ac and PzTDBA.

	TDBA-Ac			PzTDBA		
	Excitation energy (eV)	Oscillator strength	Transitions	Excitation energy (eV)	Oscillator strength	Transitions
S_1	3.6005	0.0003	H→L (87.4%)	2.9738	0	H→L (90.5%)
S_2	3.8177	0.2848	H-1→L	2.9984	0.0019	H→L+1 (89.7%)
S_3	4.4930	0.1175	H→L+4 (35.1%)<br>H→L+1 (31.9%)	3.4532	0	H→L+2 (40.7%)<br>H→L+6 (38.7%)
T_1	3.2155	0	H-1→L	2.8491	0	H→L+6 (41.5%)<br>H→L+2 (31.7%)
T_2	3.5942	0	H→L (87.3%)	2.9675	0	H→L (90.3%)
T_3	3.6211	0	H→L+5 (53.4%)<br>H→L+4 (30.1%)	2.9935	0	H→L+1 (89.7%)

Table S3 The DFT (M06-2X, 6-311g**, PCM = toluene) calculated visualized distribution of frontier molecular orbitals (HOMO-2 to LUMO+2) of TDBA-Ac and PzTDBA.

	TDBA-Ac	PzTDBA
HOMO-2	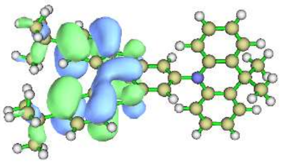	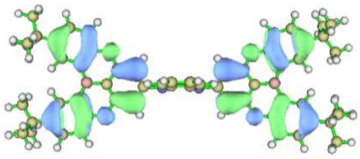
HOMO-1	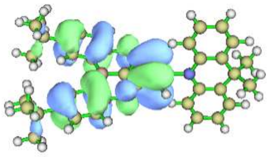	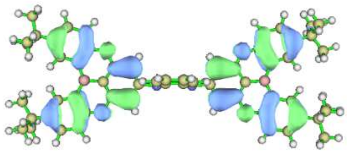
HOMO	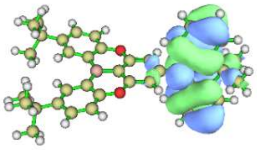	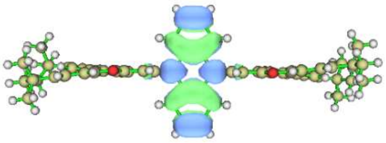
LUMO	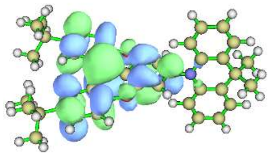	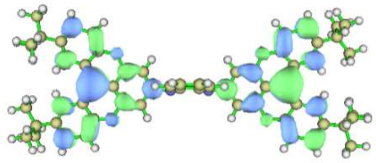
LUMO+1	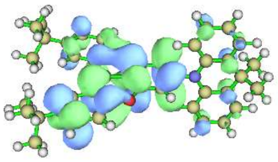	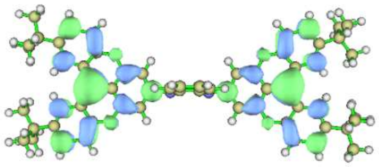
LUMO+2	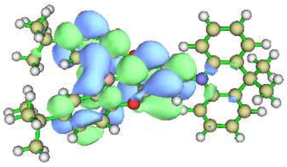	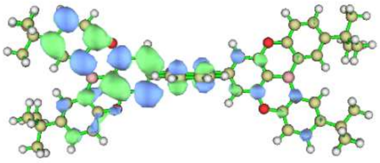

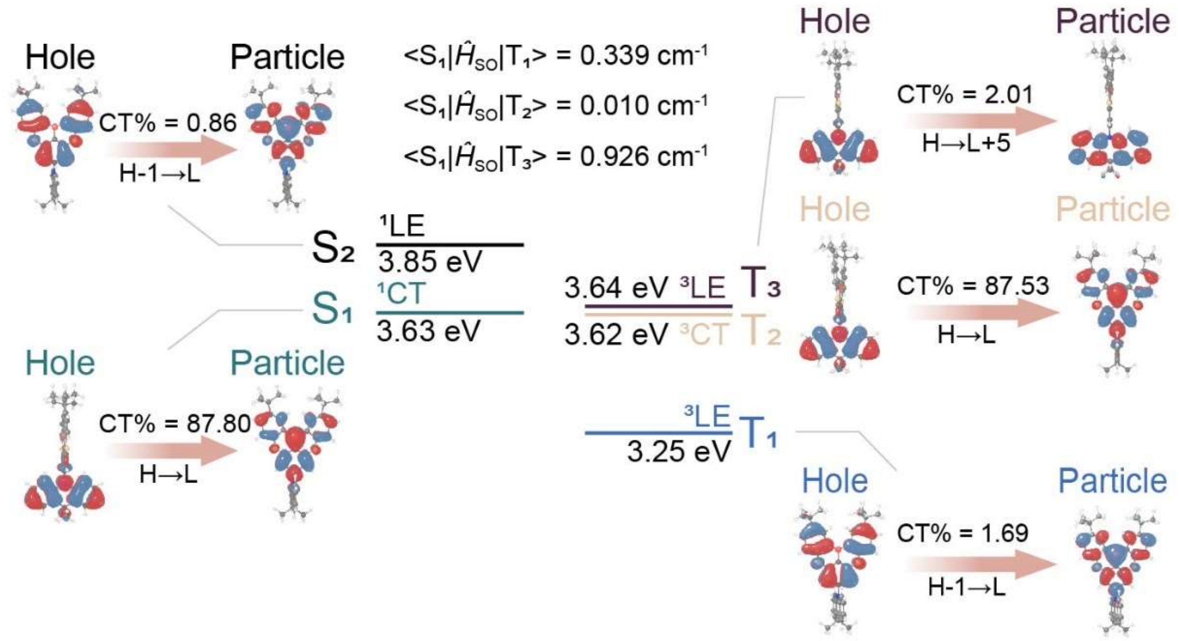

Fig. S4 Natural transition orbital (NTO) analysis and vertical energy on S_1, S_2, T_1, T_2 and T_3 states of TDBA-Ac calculated by TD-DFT (M06-2X, 6-311g**, PCM = toluene).

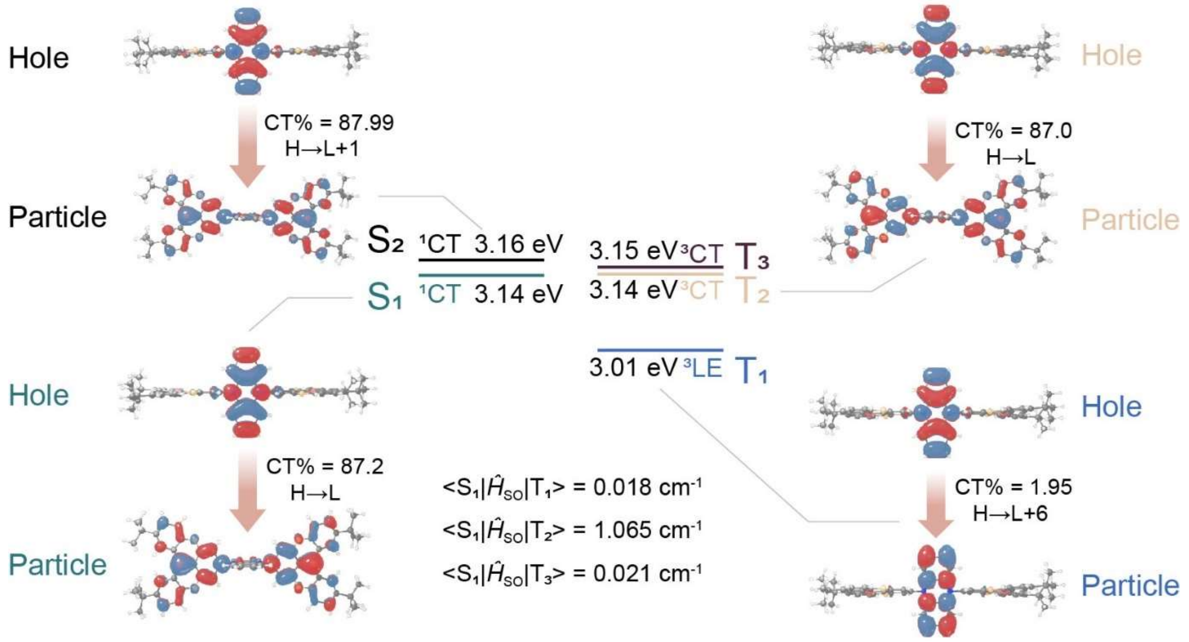

Fig. S5 Natural transition orbital (NTO) analysis and vertical energy on S_1, S_2, T_1, T_2 and T_3 states of PzTDBA calculated by TD-DFT (M06-2X, 6-311g**, PCM = toluene).

Table S4 The calculated CT parameters from hole-electron analysis of S_1 to S_3 state and T_1 to T_3 state of TDBA-Ac and PzTDBA by TD-DFT (M06-2X, 6-311g**).

		[a]Hole%	[b]Electron%	[c]Overlap%	[d]CT%	[e]S_r
TDBA-Ac	S_1	93.78	5.98	23.69	87.8	0.174
	S_2	1.55	2.41	1.94	0.86	0.697
	S_3	94.94	84.55	89.59	10.39	0.694
	T_1	1.29	2.97	1.96	1.69	0.704
	T_2	93.6	6.08	23.85	87.53	0.189
	T_3	96.37	98.39	97.38	2.01	0.840
PzTDBA	S_1	93.14	5.93	23.5	87.2	0.181
	S_2	93.14	5.16	21.92	87.99	0.179
	S_3	93.04	92.05	9.55	0.99	0.686
	T_1	94.08	96.03	95.05	1.95	0.765
	T_2	93.11	6.12	23.87	86.99	0.186
	T_3	93.11	5.20	22.00	87.92	0.182

[a]contribution percentage of TDBA (acceptor) to the hole distribution of hole-electron analysis; [b]contribution percentage of TDBA to the electron distribution of hole-electron analysis; [c]hole-electron overlap percentage of hole-electron analysis; [d]contribution percentage of CT character for specific states, LE% = 1-CT%; [e]the hole-electron overlapping indicator between 0 and 1.

Section S6. Energy gap between S_1 and T_1.

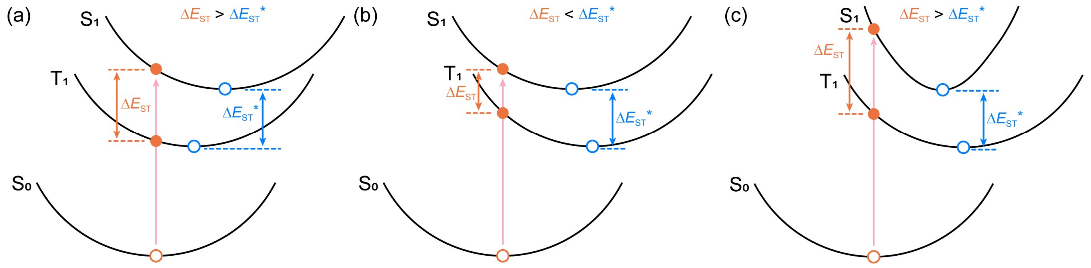

Fig. S6 Schematic diagram of vertical (ΔE_{ST}) and adiabatic (ΔE_{ST}^*) singlet-triplet energy gap of TADF emitters.

Section S7. Time-resolved Fluorescence.

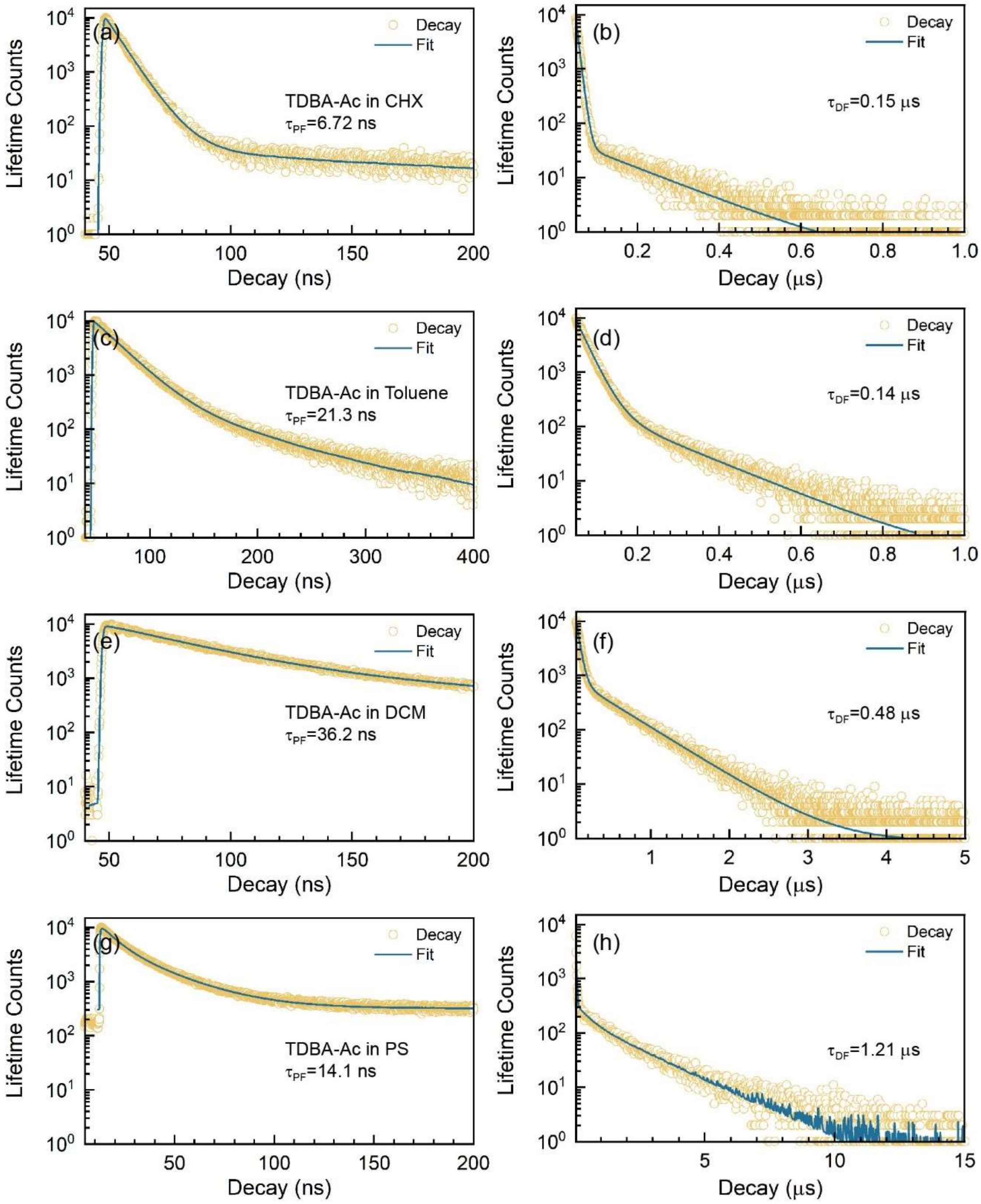

Fig. S7 Fluorescence decay kinetics of TDBA-Ac in different solutions and PS doping film upon optical excitation at 330 nm.

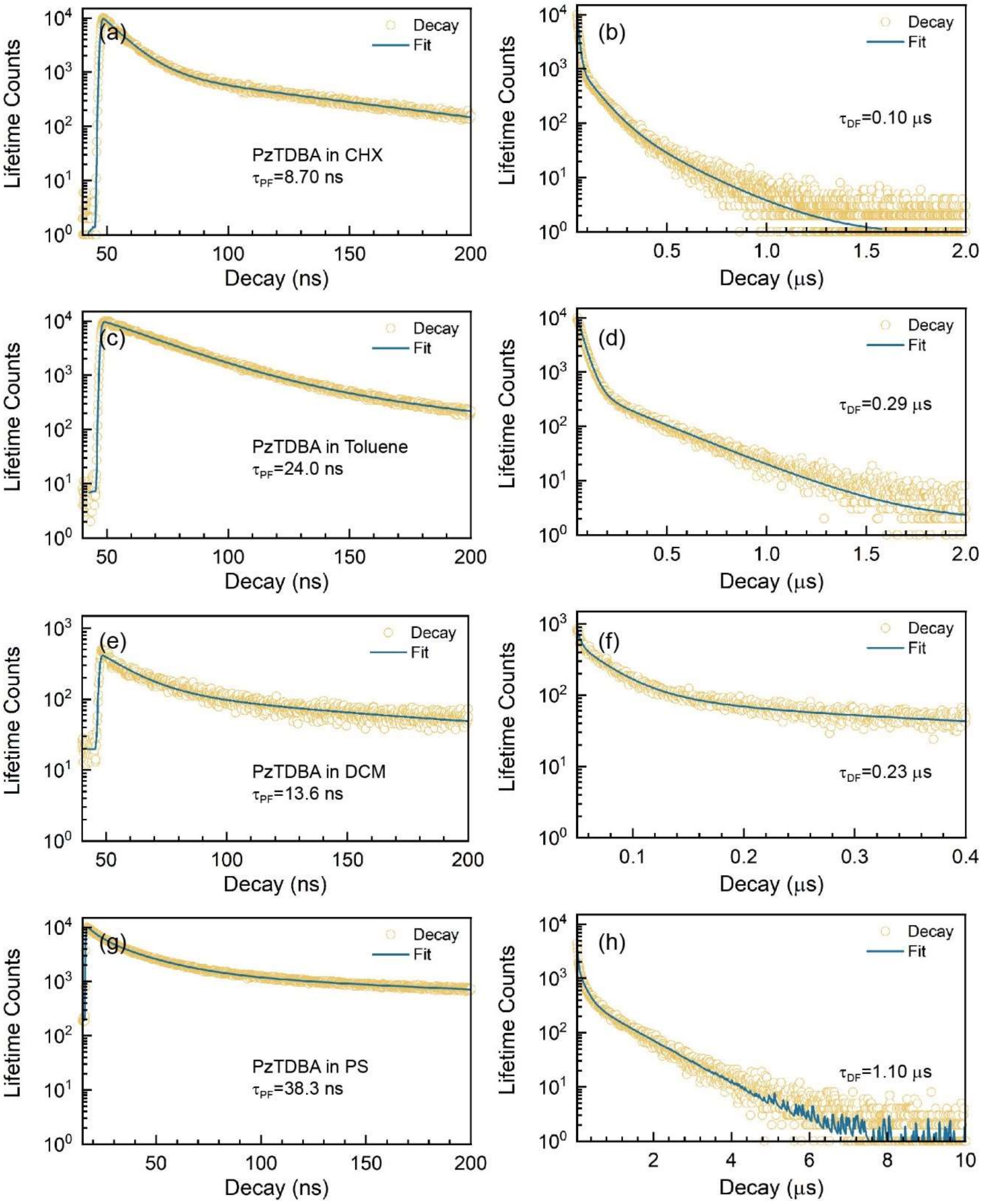

Fig. S8 Fluorescence decay kinetics of PzTDBA in different solutions and PS doping film upon optical excitation at 330 nm.

Section S8. Calculating k_{ISC} and k_{RISC}.

Table S5 Calculated k_{ISC} and k_{RISC} of TDBA-Ac and PzTDBA by Adachi's method, thermal vibration correlation function (TVCF) and semi-classical Marcus approaches.

		k_{ISC}/10^7 s^{-1}			k_{RISC}/10^5 s^{-1}		
		[a]Exp.	[b]TVCF	[c]Marcus	[a]Exp.	[b]TVCF	[c]Marcus
TDBA-Ac	CHX	8.50	1.95	7.22	18.75	<0.01	>100
	TOL	3.14	1.97	7.19	17.57	<0.01	>100
	DCM	2.15	0.33	4.99	4.98	25.29	>100
PzTDBA	CHX	10.66	<0.01	<0.01	13.42	<0.01	0.44
	TOL	3.25	<0.01	<0.01	0.52	<0.01	0.53
	DCM	/	<0.01	0.14	/	<0.01	2.81

[a] calculated by method described by Adachi *et al.*; [b] TVCF calculated by MOMAP program; [c] calculated by semi-classical Marcus approaches according to eq.6-10.

Section S9. fs-TA measurements.

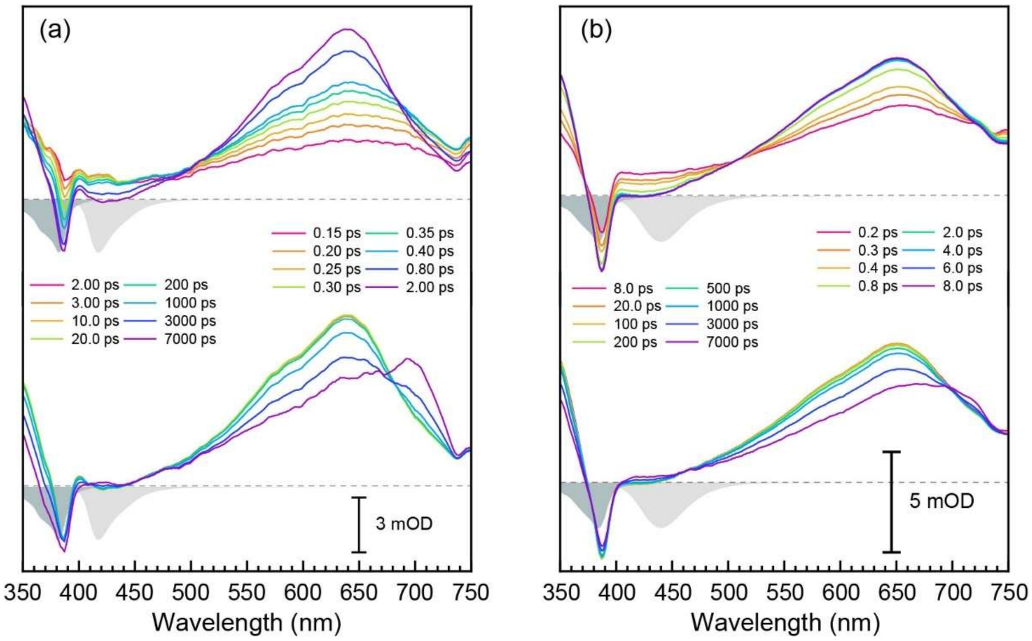

Fig. S9 Measured fs-TA spectra of TDBA-Ac in CHX solution (a) and PS doping film (b) at selected delay times upon optical excitation at 320 nm.

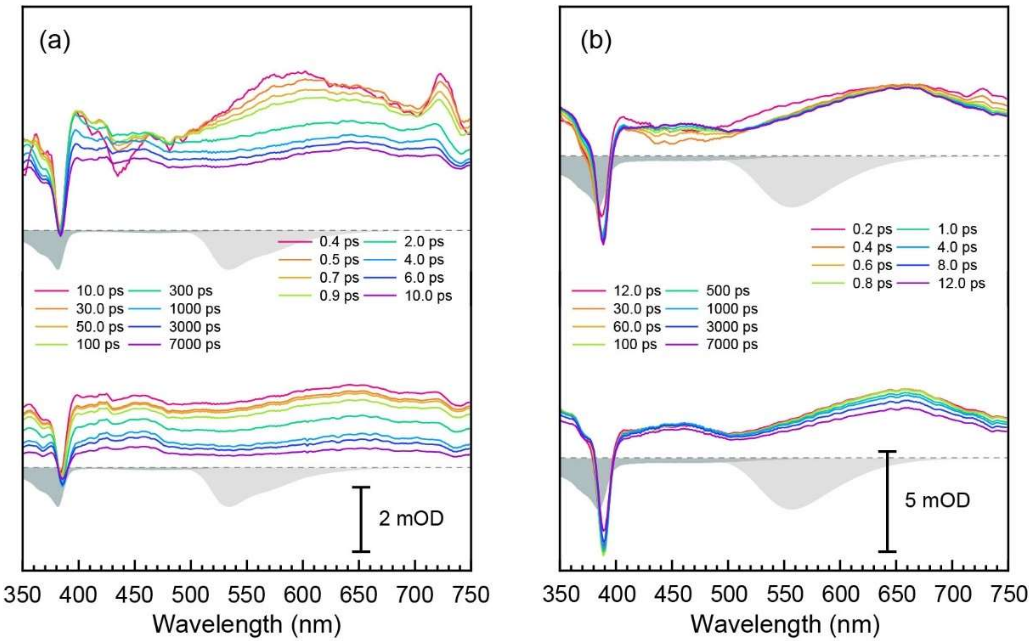

Fig. S10 Measured fs-TA spectra of PzTDBA in CHX solution (a) and PS doping film (b) at selected delay times upon optical excitation at 320 nm.

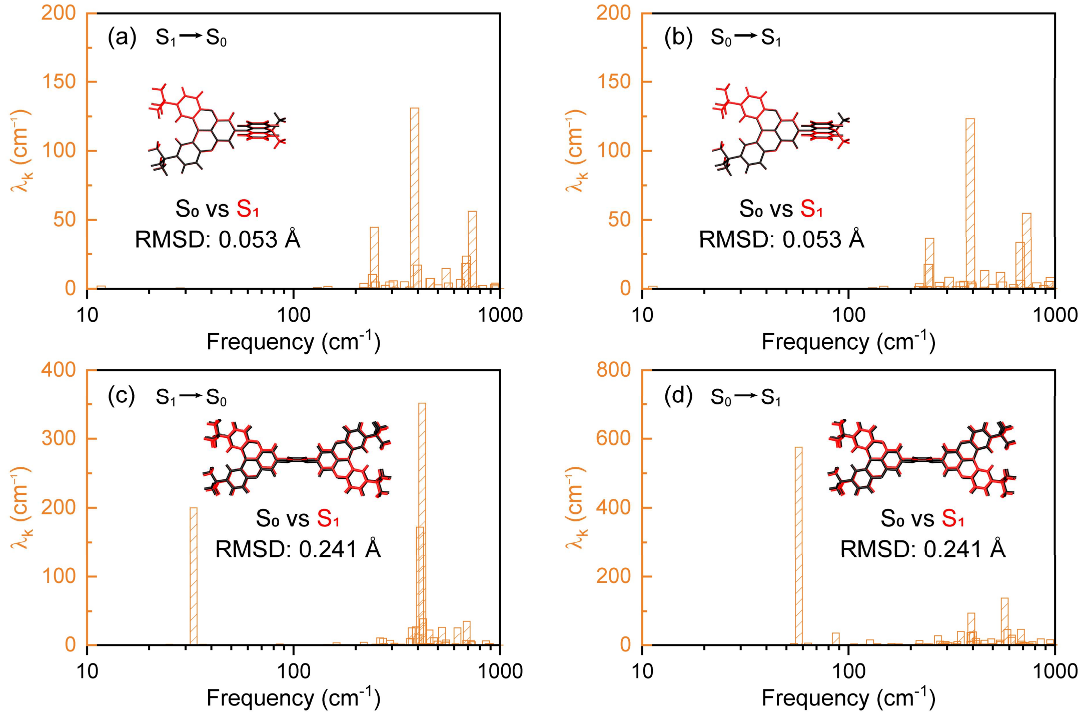

Fig. S11 Estimation of total reorganization energy ($\lambda_{S0\rightarrow S1}$ and $\lambda_{S1\rightarrow S0}$) and $RMSD_{S1/S0}$ of TDBA-Ac and PzTDBA emitters.

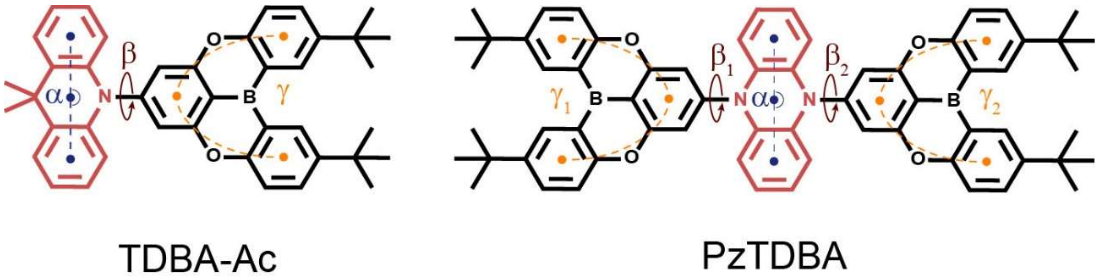

Fig. S12 Structure of TDBA-Ac and PzTDBA, a, bending dihedral angle of donor (Ac/Pz); b, donor-acceptor twisting angle; g, bending dihedral angle of acceptor (TDBA)

Table S6 The DFT and TD-DFT (M06-2X, 6-311g**, PCM = toluene) calculated optimal geometrical structures of TDBA-Ac and PzTDBA in both ground and excited-states.

	TDBA-Ac		PzTDBA	
	Front view	Side view	Front view	Side view
S_0	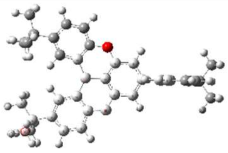	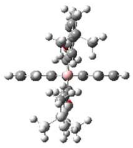	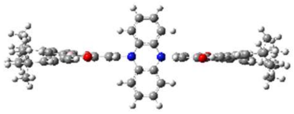	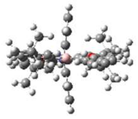
S_1	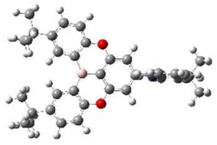	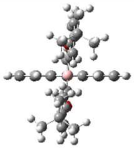	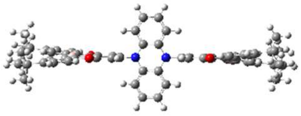	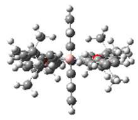
T_1	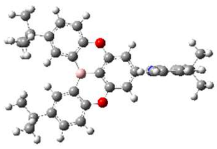	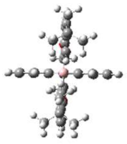	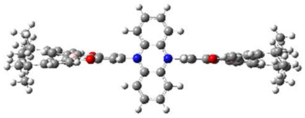	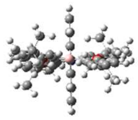

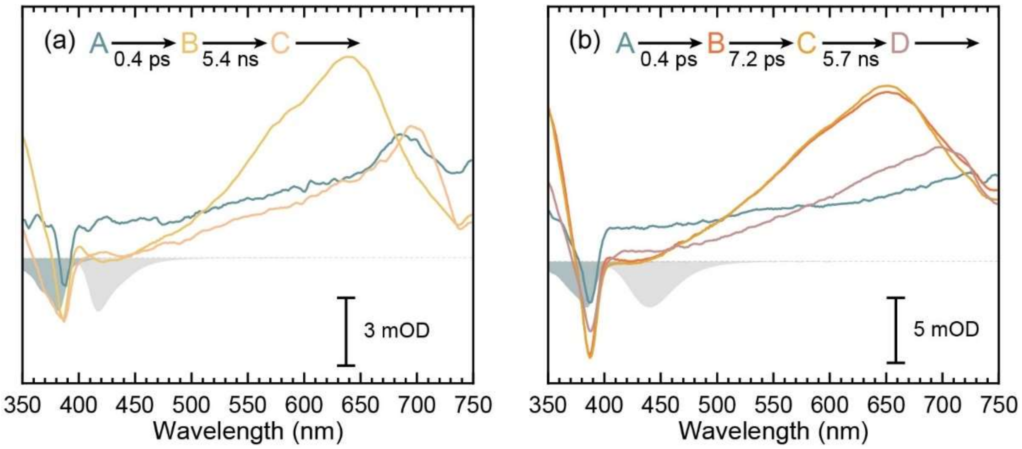

Fig. S13 The target analysis (sequential model) reconstructed species-associated spectra (SAS) of transient species of TDBA-Ac in CHX solution (a) and PS doping film (b) upon optical excitation at 320 nm.

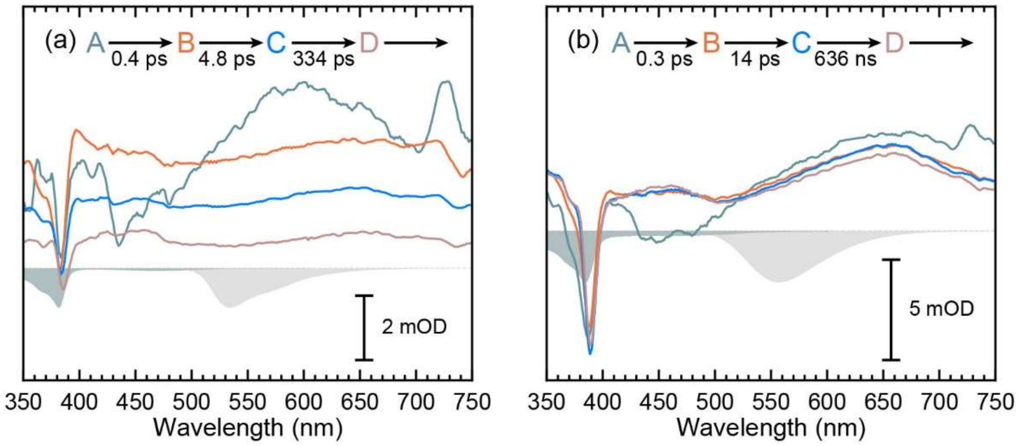

Fig. S14 The target analysis (sequential model) reconstructed species-associated spectra (SAS) of transient species of PzTDBA in CHX solution (a) and PS doping film (b) upon optical excitation at 320 nm.

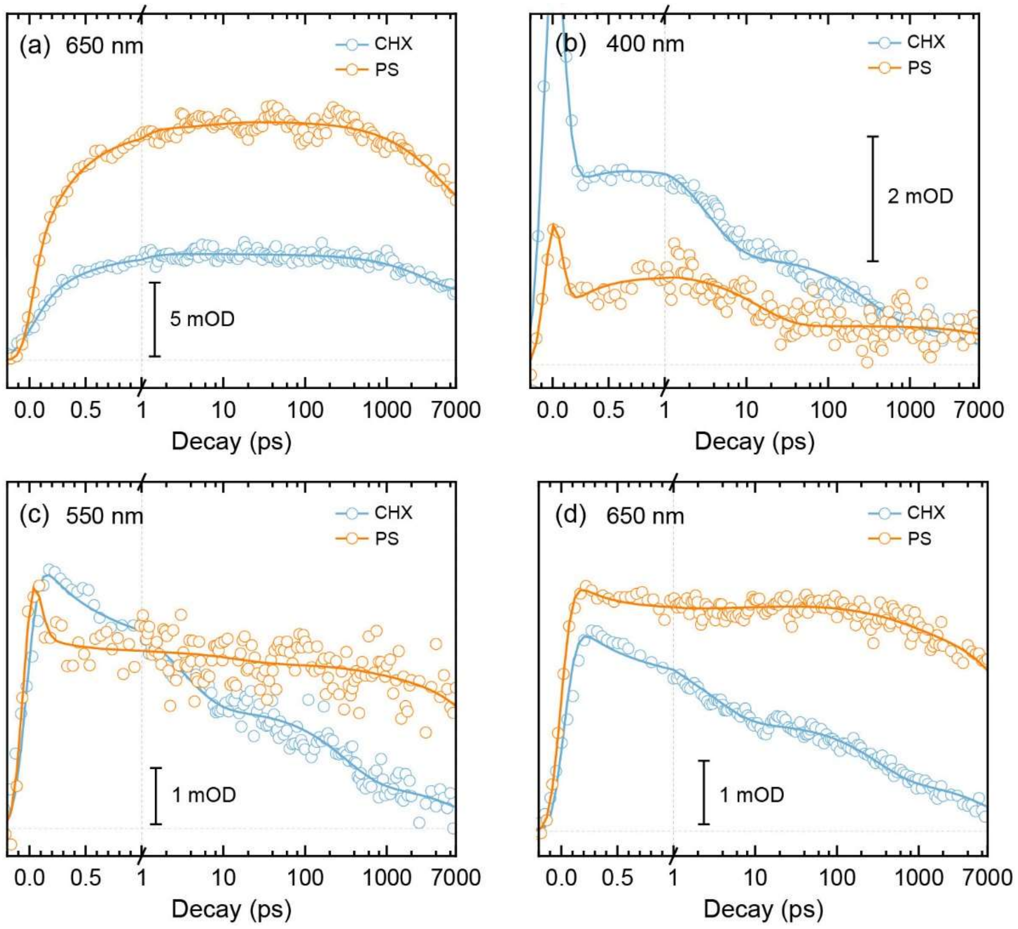

Fig. S15 Transient absorption time traces of TDBA-Ac at 650 nm (a) and PzTDBA at 400 nm (b), 550 nm (c), 650 nm (d) upon optical excitation at 320 nm in CHX solution and PS doping film; open circle: raw data, solid line: fitting

Section S10. Vibrational analysis.

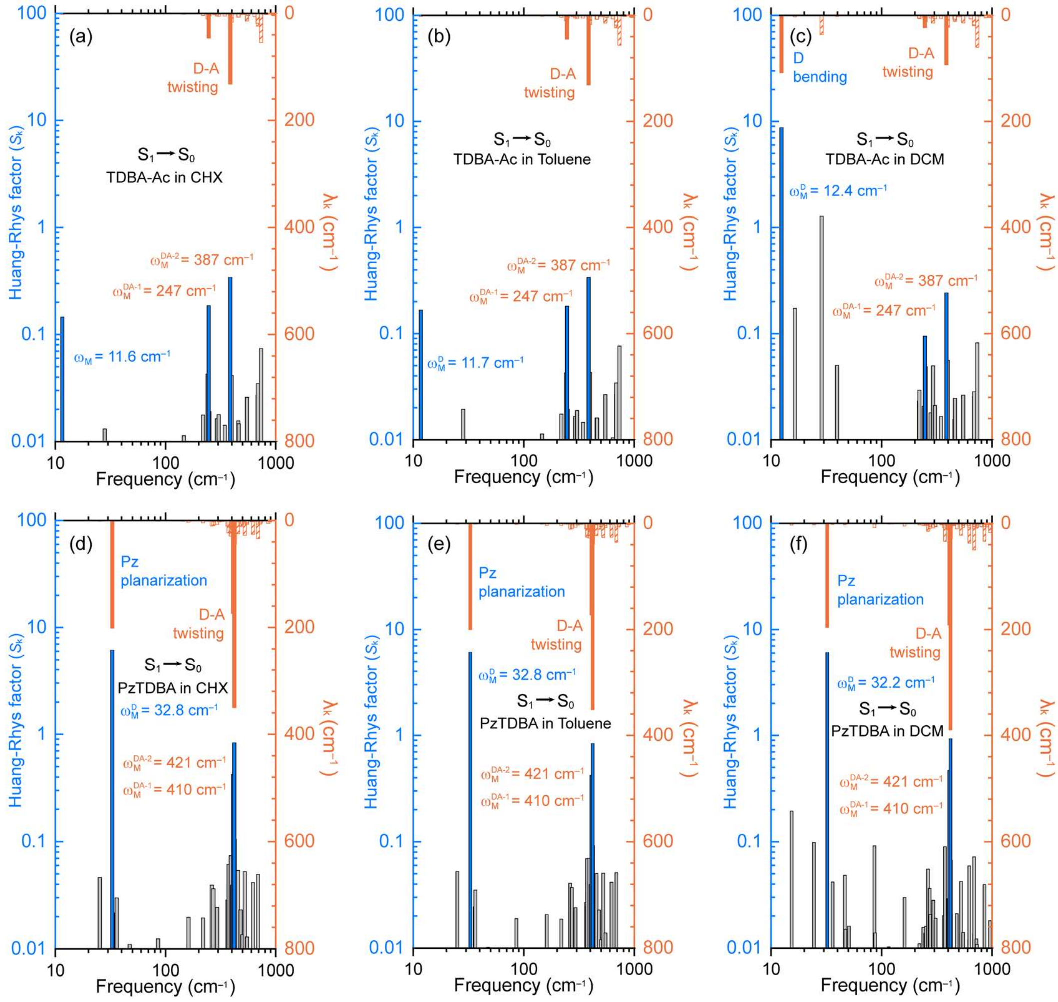

Fig. S16 Calculated HR factors and reorganization energy contribution of each vibrational modes of TDBA-Ac and PzTDBA for $S_1 \rightarrow S_0$ transition in CHX (a, d), toluene (b, e) and DCM (c, f) solutions.

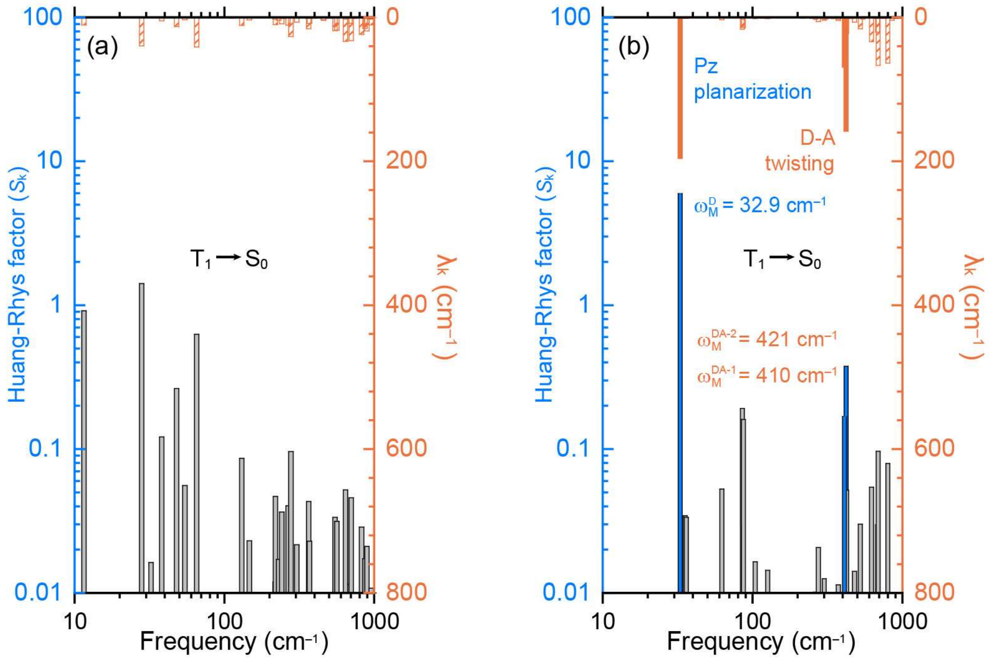

Fig. S17 Calculated HR factors and reorganization energy contribution of each vibrational modes for T_1→S_0 transition of TDBA-Ac (a) and PzTDBA (b).

Section S11. Temperature-dependent fluorescence spectra.

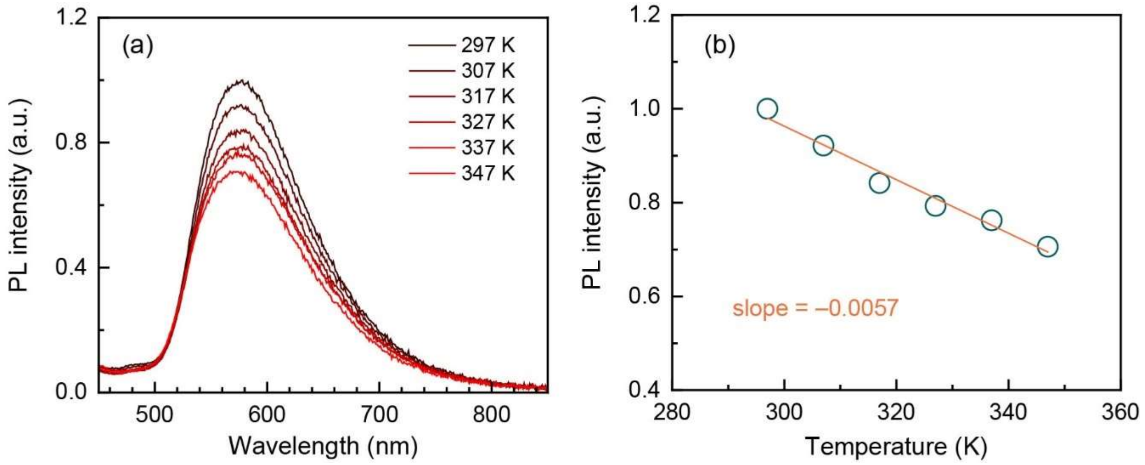

Fig. S18 (a) The temperature-dependent fluorescence spectra of PzTDBA in PEO doping film; (b) Linear fitting of the temperature-dependence of fluorescence intensity.
